# Antifungal stilbene impregnation: transport and distribution on the micron-level

**DOI:** 10.1093/treephys/tpy073

**Published:** 2018-07-10

**Authors:** Martin Felhofer, Batirtze Prats-Mateu, Peter Bock, Notburga Gierlinger

**Affiliations:** Institute for Biophysics, Department of Nanobiotechnology, University of Natural Resources and Life Sciences, Muthgasse, Vienna, Austria

**Keywords:** Confocal Raman Microscopy, extractives, heartwood formation, lipids, pine, pinosylvin, stilbene

## Abstract

The transition from the living water-transporting sapwood to heartwood involves in many tree species impregnation with extractives. These differ in amount and composition, and enhance resistance against bacteria, insects or fungi. To understand the synthesis, transport and impregnation processes new insights into the biochemical processes are needed by in-situ methods. Here we show the extractive distribution in pine (*Pinus sylvestris*) microsections with a high lateral resolution sampled in a non-destructive manner using Confocal Raman Microscopy. Integrating marker bands of stilbenes and lipids enables to clearly track the rapid change from sapwood to heartwood within one tree ring. The higher impregnation of the cell corner, compound middle lamella, the S3 layer and pits reveals the optimization of decay resistance on the micron-level. Furthermore, deposits with changing chemical composition are elucidated in the rays and lumen of the tracheids. The spectral signature of these deposits shows the co-location of lipids and pinosylvins with changing ratios from the living to the dead tissue. The results demonstrate that the extractive impregnation on the micro- and nano-level is optimized by a symbiotic relationship of lipids and pinosylvins to enhance the tree’s resistance and lifetime.

## Introduction

**Figure 1.**
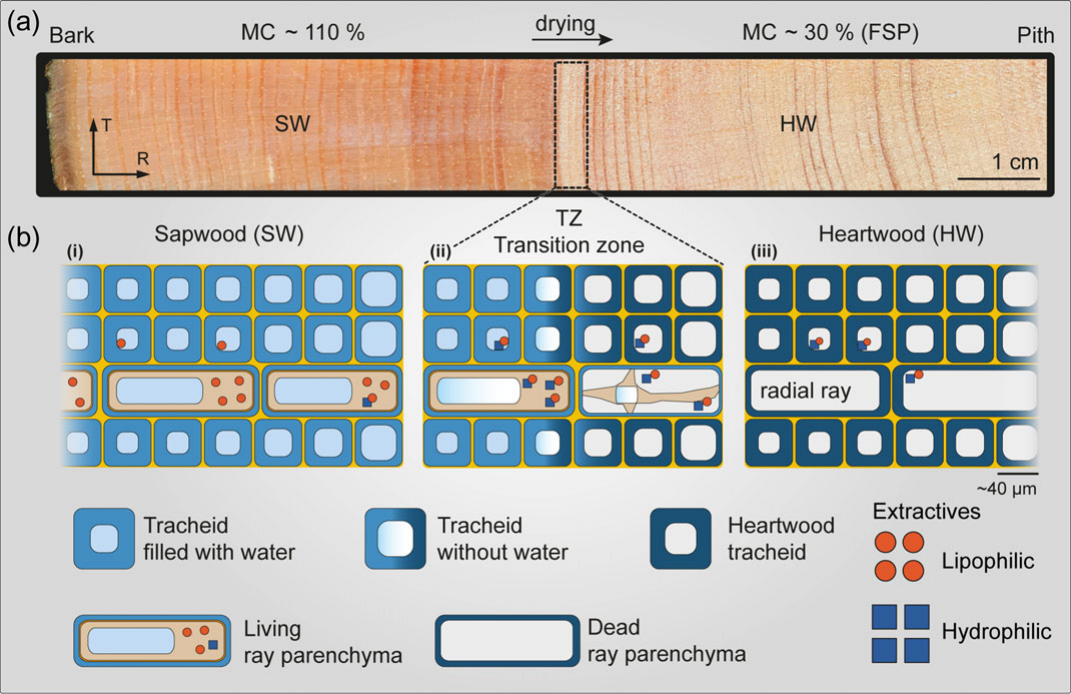
Model of heartwood formation in conifers. (a) Fresh felled pine tree with the sapwood (SW) darker due to the higher moister content (MC) than the heartwood (HW). (b) Sketch of the three main areas: (i) sapwood with a higher moisture content and living parenchyma cells; (ii) transition zone where the radial rays undergo a programmed cell death and the tracheids dry out; and (iii) heartwood tracheids impregnated with heartwood constituents and lower moisture content (around fiber saturation point, FSP). Sketch inspired by Nakada and Fukatsu (2012).

Wood is part of the natural carbon cycle and is finally degraded by bacteria, insects and fungi (Fromm 2013). To enhance resistance to biotic and abiotic decay and to increase mechanical stability, in the course of lignification and heartwood formation trees impregnate their tissues with highly polymerized phenolic compounds (Figure 1) (Hillis 1987, Fengel and Wegener 2003, Bentz et al. 2017). Today, more than 8000 plant phenolics are known and are classified into two main groups, either flavonoid or non-flavonoid compounds (Strack and Wray 1994). These plant-associated substances are currently a hot topic in a wide range of research areas (Savio et al. 2016, Berman et al. 2017, Adebooye et al. 2018, Arif et al. 2018, Donado-Pestana et al. 2018, Halake et al. 2018). In particular, the 3,4′,5-trihydroxystilbene, known as resveratrol, has attracted a lot of research attention (Bostanghadiri et al. 2017). Many plants provide these nutraceuticals including wood (Burns et al. 2002, Dubrovina and Kiselev 2017, Sebastia et al. 2017), blueberries, red grapes (see Figure S1 available as Supplementary Data at *Tree Physiology* Online) and are thus also present in red wine at high concentration (0.2–14.3 mg l^−1^) (Stervbo et al. 2007). This is suggested to be the solution to the ‘French paradox’ (Singh et al. 2013, Ma et al. 2014). Quite similar to the ‘French paradox’, the answer to the question, ‘Why do some trees live longer than others?’ is related to the presence of extractives. In fact, the oldest tree (5000+ years) (Brunstein and Yamaguchi 1992) still alive belongs to the species *Pinus longaeva* (see Figure S1 available as Supplementary Data at *Tree Physiology* Online) and has a high amount of extractives (stilbenes/pinosylvins) similar to resveratrol (Schulman 1958). Extractives do not solely allow the living tree to resist the attack of insects and microorganisms but also give protection to the processed wood. For example, the pagoda of the Horyuji temple (see Figure S1 available as Supplementary Data at *Tree Physiology* Online) in Japan is known as the oldest man-made wooden structure in the world, showing the ability of wood as a construction material to resist decay for more than 1300 years.

The in-vivo impregnation with phenolics takes place on the micro- and nano-level as these waterproofing components are incorporated in the cell wall and also between the different cells (Micic et al. 2001, Vanholme et al. 2010). The final step in the life cycle of the living xylem is the transition from sapwood into heartwood and is considered as a secondary differentiation process (Figure 1). Therefore in mature trees, the xylem can be divided into (i) the outer sapwood, a conductive tissue with living parenchyma cells and (ii) the heartwood consisting of ‘dried out’ cells, in many species recognized by a darker color due to additional impregnation with phenolic compounds. In-between, within the so-called transition zone or intermediate wood, the following transformation takes place (Figure 1) (Bergström 2000, Bergström 2003). The radial parenchyma cells, which extend radially from the phloem inwards (Schultz and Nicholas 2000, Nakaba et al. 2008, 2012) die (Figure 1b), accompanied by a decrease in moisture content and a depletion of starch and triglycerides (Hillis 1987, Pallardy 2008). Furthermore, darkening of the wood and accumulation of extractives in the xylem is observed (Mayer et al. 2006, Taylor et al. 2011, Tomppo et al. 2011). Radial rays play a major role in the biosynthesis, storage and/or transport of extractives and/or their precursors, especially in the transition zone (Lim et al. 2016). Transport of water between neighboring tracheids, vessels and parenchyma cells is accomplished through the pits (Choat et al. 2008). Krahmer et al. (1970) reported the presence of extractives in the torus of heartwood pits by using transmission electron microscopy, but no information about the chemical nature of these deposits was given. Furthermore, there are some anatomical changes related to the decrease in moisture content such as pit aspiration in conifers (Petty 1972) and the formation of tyloses in dicots (Kitin et al. 2010, Murmanis 1975).

Depending on the trees species, heartwood extractives of different composition (terpenes, flavonoids, lignans, stilbenes or tropolones, among others) and in various amounts are deposited (Hillis 1987) under a tight genetic and environmental control (Lim et al. 2016). In some species, extractives are hardly found (e.g., spruce 0.9–1.5%) (Willför and Holmbom 2004), whereas in others quite high amounts are detected (e.g., larch up to 30%) (Gierlinger et al. 2004). The content of polyphenols is generally higher in the heartwood of old trees than in that of young trees and decreases from the periphery towards the central portion of the stem (Gierlinger and Wimmer 2004). Furthermore, large quantities have been observed in injured wood, knot wood, resin canals, needles and cones (Willför et al. 2003, Zulaica-Villagomez et al. 2005). As the extractives are responsible for natural durability, different grades are achieved depending on species, age and position within the stem (Schultz and Nicholas 2000, Taylor et al. 2002, Gierlinger and Wimmer 2004).

The yield of different extractives depends on the extraction method, polarity of the solvent, temperature and time (Xu and Chang 2007, Fang et al. 2013). For example, pine extractives can be divided into a hydrophilic (stilbenes and lignans) and a lipophilic part (resin acids, long chain fatty acids and triglycerides) (Figure 1b). By sequentially extracting pine heartwood chips with non-polar and polar solvents different extracts can be obtained (Lu et al. 2016). For example, the extraction with a polar solvent recovers the pinosylvins with high purity (Fang et al. 2013). Up to now, only a few studies dealing with stilbene impregnation of wood have been conducted. Already in 1943 (Rennerfelt) and 1945 (Rennerfelt), it was observed that sapwood impregnated with pinosylvins became more stable against fungal degradation. Later on, Celimene et al. (1999) impregnated maple with a mixture of pinosylvins, and Seppanen et al. (2004) birch and aspen, to reduce fungal decay. Recently, the textile industry has become interested in polyphenol impregnation to inhibit the bacterial growth (Sanchez-Sanchez et al. 2017). Furthermore, novel food packaging (e.g., Busolo and Lagaron (2015)) and medical (e.g., resveratrol in diabetic wound area (Gokce et al. 2017)) applications have been developed.

Chaffey (2002) asked ‘Why is there so little research into the cell biology of the secondary vascular system of trees?’ and addressed the lack of funding, techniques and the imparity between primary and secondary cell wall research. Heartwood extractives have been mainly investigated by wet-chemical and chromatographic methods (Ekeberg et al. 2006) and recently also genetic approaches (Paasela et al. 2017). To investigate extractives in context with microstructure, TOF-SIMS imaging has been applied in *Cryptomeria japonica* trees and showed that the extractives tend to accumulate near the radial rays (Saito et al. 2008, Kuroda et al. 2014). Recently Belt et al. (2017) showed the potential of Confocal Raman Microscopy (CRM) to follow the extractive distribution in sap- and heartwood of Scots pine. On the micro-level, conglomerating phenolics were reported in the lumen as well as a higher amount of phenolics in the compound middle lamella (CML) and cell corner (CC).

Confocal Raman Microscopy relies on the inelastic scattering of incident monochromatic light (hv_0_), wherein the energy of the scattered light changes upon interaction with molecular vibrations (e.g., bond stretching, rotation and torsion) of the sample, resulting in either Stokes (hv_S_) or anti-Stokes (hv_aS_) scattering and thus revealing the nature of its components (Colthup et al. 1990). Important new insights on the molecular structure of wood polymers, especially cellulose and lignin, could be gained (Agarwal 2005, 2006, 2014, Agarwal et al. 2016). Confocal Raman Microscopy applied in area mapping mode gives molecular fingerprints (Raman spectrum) at every pixel with a high lateral resolution (~300 nm) and enables monitoring of the chemical changes at the microscale in the context of the anatomical wood structure in the native state (e.g., Agarwal 2006, Gierlinger 2011, Gierlinger et al. 2012).

The main objective of this study was to monitor heartwood formation with CRM exactly in the transition zone of native pine samples (*Pinus sylvestris*) and compare it with an in-vitro impregnation. The experimental design focuses on chemical changes occurring in tracheid cell walls and radial rays with the aim to understand the transport pathways of natural and in-vitro extractives impregnation and wood functionalization.

## Materials and methods

### Plant material

Never-dried wood samples were obtained from pine (*P. sylvestris*) trees sampled in a forest in upper Austria (48°31′50′N, 13°55′44′E). The sample discs (~50 mm thick) were cut at breast height 130 cm above ground. The macroscopic delineation of heartwood and sapwood was determined in green wood by the color difference due to the different moisture content in both regions and the disc was immediately frozen at −20 °C to preserve the native state (especially the moisture content). Rectangular pieces were cut out from sapwood and heartwood in both north and south cardinal directions of the disk (see Table S1 available as Supplementary Data at *Tree Physiology* Online) to record the most important wood characteristics (e.g., age, moisture content and density). For rotary microtomy T-shaped blocks (25 × 15 × 20 mm, radial × tangential × axial) were cut at the sapwood–heartwood transition zone from the south cardinal direction to avoid overpressure in the area of interest during the sectioning.

### Wood extraction

Air-dried pine heartwood chips from the same tree were splintered with a chisel, ground with a coffee mill and dried in an oven (103 °C). The dried wood was conditioned to room temperature in a desiccator and the initial dry weight was recorded. Crude extractives were obtained using a Soxhlet extractor (100 ml) with 23 g dry pine heartwood chips. To extract sequentially, lipophilic extractives were first collected with *n*-hexane (≥95%, Carl Roth GmbH + Co. KG, Karlsruhe, Germany); thereafter the stilbene-rich fraction (hydrophilic) was extracted with ethanol (≥99.5%, Carl Roth GmbH + Co. KG, Karlsruhe, Germany); both cycles ran for ~18 h. To get the pure extract, the EtOH was removed under vacuum (90 mbar) by rotary (150 rpm) evaporation at 40 °C. The residue (900 mg) was dissolved in 30 ml of EtOH to a concentration of 30 mg ml^−1^ and stored at −20 °C in a freezer.

### Impregnation and sectioning

Fresh wood blocks (7 × 7 × 20 mm^3^; R × T × A) were collected from spruce sapwood and oven-dried for 12 h at 60 °C and for 1 h at 103 °C. Then, they were conditioned in a desiccator to room temperature under vacuum (10 mbar) to get rid of the air in the microstructure (see Figure S2a and b available as Supplementary Data at *Tree Physiology* Online). The blocks were grouped into ‘control’ and ‘impregnated’ before being immersed either in pure EtOH or crude-extract-EtOH solution (30 mg ml^−1^). Each block was placed in a vial with 6 ml extract solution under ambient pressure for 20 h (see Figure S2a and b available as Supplementary Data at *Tree Physiology* Online). Thereafter the blocks were repeatedly impregnated in the vacuum (100 mbar). One cycle took 10 min at room temperature and at least three cycles were carried out. After impregnation, the blocks were dried at 103 °C and the kiln dry weight was recorded. Before sectioning, the blocks were immersed with Deuterium (D_2_O) under vacuum to reduce the overlap of the OH and CH stretching bands during the Raman measurements.

### Microsectioning

The T-shaped block was tightly clamped in a rotary microtome (RM2235, Leica Biosystems Nussloch GmbH, Wetzlar, Germany) with an orientation perpendicular to the main fiber axis. Disposable microtome blades (N35HR Blade 35°, Feather, Osaka, Japan) were used to perform 10–20 μm thick transverse sections. During the cutting process, only D_2_O was used to avoid drying of the specimen and maintaining the native water content. The thin sections were placed on a standard glass slide with a drop of D_2_O, covered with a glass coverslip (0.17 mm thick) and sealed with nail polish. The sapwood–heartwood boundary was marked on the bottom of the slide and measured immediately or kept frozen until CRM measurement.

### Confocal Raman Microscopy

Raman spectra were acquired with a Confocal Raman Microscope (alpha 300RA, WITec GmbH, Ulm, Germany) equipped with a piezo motorized scan stage (x–y–z). The excitation light source was a linear polarized (0°) coherent compass sapphire green laser λ 532 nm (WITec, Ulm, Germany) focused through a coverslip-corrected 100× oil objective (NA 1.4, Carl Zeiss, Jena, Germany). The Raman scattering signal was collected by the same objective, delivered by an optic multifibre (Ø = 50 μm) to the spectrometer (600 g mm^−1^ grating, UHTS 300 WITec) and finally recorded by a CCD camera (Andor DU401ABV, Belfast, UK). The orientation of the sample with respect to the laser polarization (the radial direction within the *y*-axis of the table) was kept constant during all measurements. All Raman scans were taken with a lateral resolution of 0.3 μm by acquiring at every pixel one spectrum with an integration time of 0.08 s and a laser power of 30 mW. The control Four (WITec) acquisition software was used to set experimental parameters for hyperspectral image acquisition. With the same instrument, reference spectra of fatty acids (oleic, linoleic and glyceryl trilineolat), abietic acid, pinosylvin (PS), pinosylvin monomethyl ether (PSMME), pinosylvin dimethyl ether (PSDME) and resveratrol, all purchased from Sigma-Aldrich (Vienna, Austria), were measured. The detailed assignment of all references is listed in the Assignment Tables S1–S8 (available as Supplementary Data at *Tree Physiology* Online). For further information, these reference spectra can be also found on the webpage www.bionami.at.

### Spectra processing and data analysis

Raman data analysis was performed with Project FOUR (WITec GmbH, Ulm, Germany) and ImageLab (EPINA GmbH, Pressbaum, Austria) software. The extracted spectra were analyzed with Opus 7.5 software^TM^ (Bruker, Rheinstetten, Germany). Before the Raman images were generated based on integration of specific bands, a cosmic ray removal filter (spike half-width 2) was applied. Based on the integrated images, average spectra of distinct areas of the samples (e.g., CC, cell wall and deposits) were obtained by drawing areas of interests or using an intensity threshold. The principal component analysis (PCA) in the spectral range 200–3170 cm^−1^ was carried out with the software OriginPro 9.1 (OriginLab Corporation, Northampton MA, US).

## Results and discussion

### Visualization of extractives in context with the microstructure

**Figure 2.**
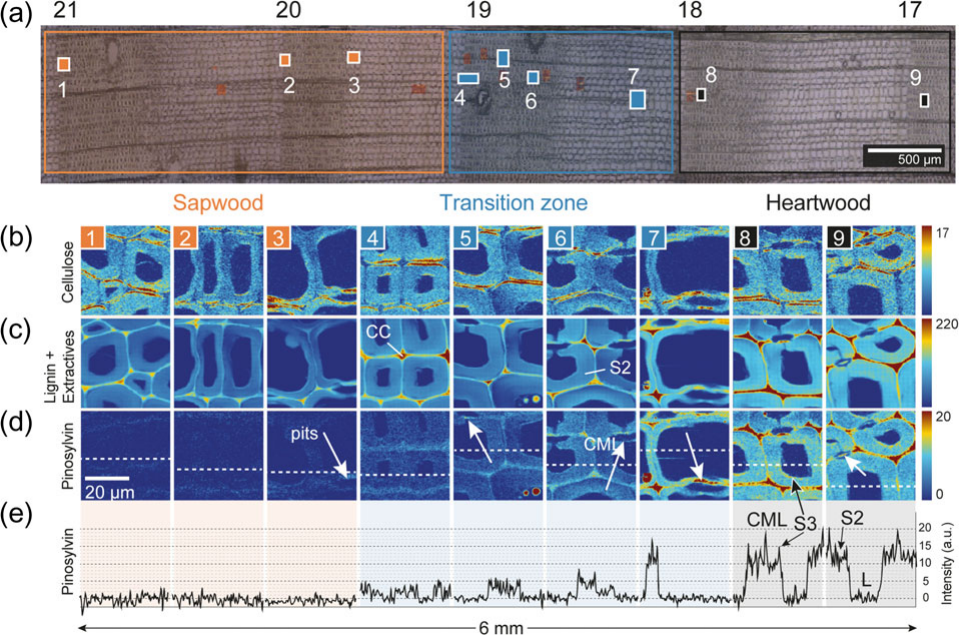
In-situ Raman mapping of pine tracheids during heartwood formation. (a) Bright field image of a transverse microsection of a fresh pine stem from sapwood to heartwood (17–21st annual ring counted from pith to bark). Raman measurement areas are marked by the numbered rectangles (1–9). (b–d) Raman images (40 × 40 μm) based on bands for (b) cellulose (1076–1110 cm^−1^), (c) phenolic compounds (1556–1694 cm^–1^) and (d) pinosylvins (982–1011 cm^–^^1^, 1,3,5-substituted aromatic ring). (e) The graph is plotted as an intensity (997 cm^–1^) profile along the white dotted lines at d. Legend: CC, cell corner; CML, compound middle lamella; S2 and S3, secondary cell walls; L, Lumen.

Raman mappings were acquired in-situ with a lateral resolution of 300 nm along five annual rings to reveal the nature and distribution of the extractives in pine (*P. sylvestris*) (Figure 2a). Based on integration over characteristic bands for cellulose, phenolic compounds (including lignin) and pinosylvins (PS, PSMME and PSDME) it was possible to follow the distribution of these components (Figure 2b–d). The cellulose Raman images (Figure 2b) depict the cell wall and show a rather constant intensity along sapwood and heartwood (small changes come from slight changes in focal plane or uneven sample surface). The S1-layer with a higher microfibril angle is emphasized in the radial direction because the integration range included the 1095 cm^−1^ band, which has been shown to become higher with high cellulose microfibril angle (Gierlinger et al. 2010). On the contrary, the intensity related to the aromatic ring stretching vibration from the phenolic compounds increases clearly towards the heartwood (Figure 2c). Although this strong 1600 cm^−1^ band is usually attributed to lignin, it is known to be typical for many different aromatic components. Reference spectra from characteristic pine heartwood extractives indeed clearly show that also pinosylvins contribute to the intensity of this band (details in Figure S3 available as Supplementary Data at *Tree Physiology* Online). Besides two strong significant bands at 1637 cm^−1^ (assigned to C = C double bond conjugated with two aromatic rings) and 997 cm^−1^ (assigned to the 1,3,5-substituted aromatic ring bending) are present, which have already been used as marker bands for pinosylvins in pine (Bergström et al. 1999, Bergström 2003, Nuopponen et al. 2004*a*, 2004*b*, Belt et al. 2017) and are also herein used to follow the pinosylvins on the micro-level.

For the characteristic pinosylvins band at 997 cm^−1^, no signal was observed in the sapwood (Figure 2d, images 1–3), but a rapid increase was seen within one annual ring in the transition zone (Figure 2d, images 4–7). Within the heartwood, the highest intensity (dark red) is found in the CC, CML, S3 layer, within the pit membrane and in various deposits in the lumen (Figure 2d). Figure 2e shows the intensity profile of the pinosylvins band at 997 cm^−1^ along the dotted virtual line depicted on the pinosylvin images (Figure 2d). The intensity profile shows clearly that extractive accumulation starts abruptly in a narrow area of approximately one annual ring (19th annual ring) as the intensity of the 997 cm^–^^1^ band raises from 5 to 15 CCD counts between the 19th and 20th annual ring (Figure 2e, images 4–7), whereas no pinosylvins are detected before (intensity ~0 a.u.). The higher accumulation in the CML becomes again clear in the intensity profile. Moreover, a slightly higher content in the S3 layer compared with the S2 layer, described by a bow-shaped intensity profile with three main peaks (Figure 2e, image 8) is visible: the intensity is high in the S3 layer of the secondary cell wall, decreases in the thick S2 to medium values and peaks to a maximum at the CML. Both layers (CML, S3) are tiny layers and close to the limit of the spatial resolution of the instrument, but still, a higher content can be detected (Figure 2e, images 8 and 9).

### Spectral signatures of heartwood formation

**Figure 3.**
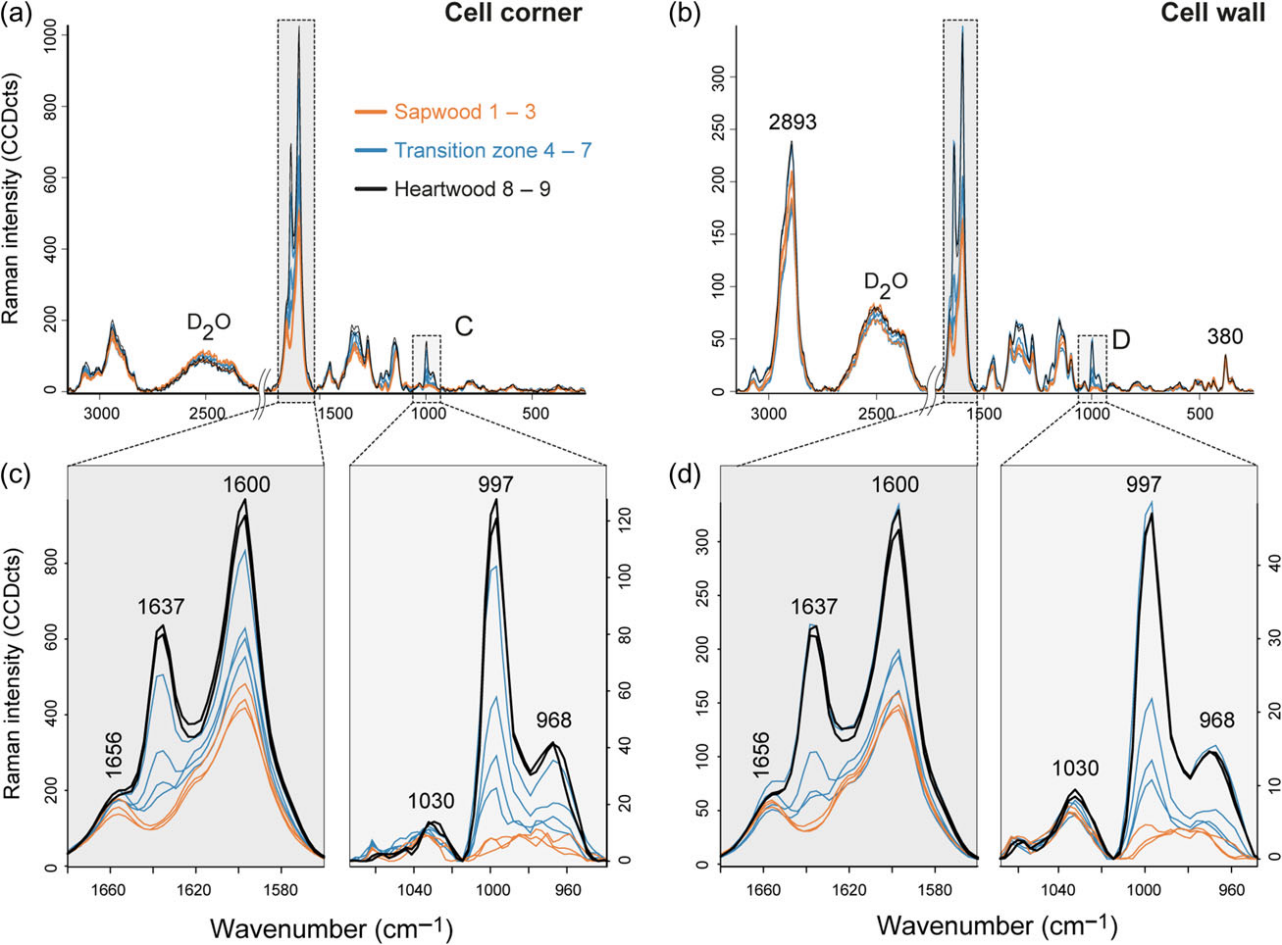
How heartwood differs—spectral view of heartwood formation. (a) Average spectra of the cell corner and (b) cell wall from sapwood, transition zone and heartwood of pine and (c, d) a zoom into the two most important aromatic wavenumber regions for pinosylvins.

To examine the chemical changes and composition during heartwood formation within the secondary cell wall and the CC selectively, average spectra were extracted. The detected Raman intensity of the most prominent phenolic bands (1600 and 1637 cm^−1^) was threefold higher in the CCs (Figure 3a) than in the cell wall (Figure 3b, note different *y*-axis scale), in which also characteristic bands for cellulose (e.g., 380 and 1095 cm^−1^) were observed as well as a higher D_2_O band. Comparing the sapwood (orange spectrum), transition zone (blue spectrum) and heartwood spectra (black spectrum) it became clear that the bands found to be characteristic for pinosylvins (1600, 1637 and 997 cm^−1^) are the ones that increased significantly, both in the CC and in the cell wall. A zoom into these spectral regions (Figure 3c and d) clearly depicted the absence of the band at 1637 cm^−1^ and 997 cm^−1^ in sapwood and the continuous increase of these bands from the transition zone to the heartwood (compare again the intensity scale). As the 1656 cm^−1^ band stays at a rather constant level (despite a slight increase due to the influence of the increasing neighbored 1637 cm^−1^ band, Figure 3c and d), it can be concluded that most of the increase of the 1600 cm^−1^ band is due to the increase of the extractives and not of lignin. Thus in heartwood studies and/or wood impregnated with extractives, the lignin marker band at 1600 cm^−1^ has to be taken with care for lignin quantification.

Discriminating between PS and PSMME is possible based on the two bands neighboring the strong 997 cm^−1^ band, as these Raman bands are not influenced by cellulose and lignin. Especially, the 968 cm^−1^ (trans CH wag of C = C) (Colthup et al. 1990) band is not present in PS, while the 1030 cm^−1^ band is higher in PS than in PSMME (Figure S3 available as Supplementary Data at *Tree Physiology* Online). Belt et al. (2017) described a doublet (994 and 998 cm^−1^) for the discrimination between PS and PSMME. However, the question is whether this tiny wavenumber difference is enough to discriminate between both substances in wood. These bands arise from vibration 12 of the benzene ring (Varsanyi 1969, Colthup et al. 1990) and are only present in mono-, meta- and sym-tri substituted rings. Pinosylvins show these two bands because both rings will exhibit this vibrational mode. However, there will be a difference between the two rings due to the different nature of the substituents (OH, OMe). Changing the environment of the rings (hydrogen-bonding etc.) can also induce changes in the frequency of this mode, which means that the split will not always be observed, rendering this feature rather useless for stilbene identification. Therefore, in the present study, PSMME will be differentiated from PS, PSDME and resveratrol by the aforementioned 968 cm^−1^ band, which is only detectable in the PSMME spectrum (see Figure S3 available as Supplementary Data at *Tree Physiology* Online).

The 968 cm^−1^ band clearly arises in the transition zone and shows a continuous increase towards the sapwood and transition zone. From this, it can be concluded that PSMME is strongly contributing to the extractive signal. The weaker 1030 cm^−1^ band is also slightly increasing and thus PS is involved. Nevertheless, the latter one is in general weaker and not completely free from lignin contribution as it is already present in the sapwood (Figure 3c and d).

Summarized, the average spectra extracted from CC and cell wall in sapwood, transition zone and heartwood indicate that the main difference was the increase of phenolic components, identified as pinosylvins and mainly PSMME (Figure 3). The spatial distribution is similar to that for lignin as higher amounts of pinosylvins were deposited in the CCs than in the cell wall.

### Detection of various deposits

**Figure 4.**
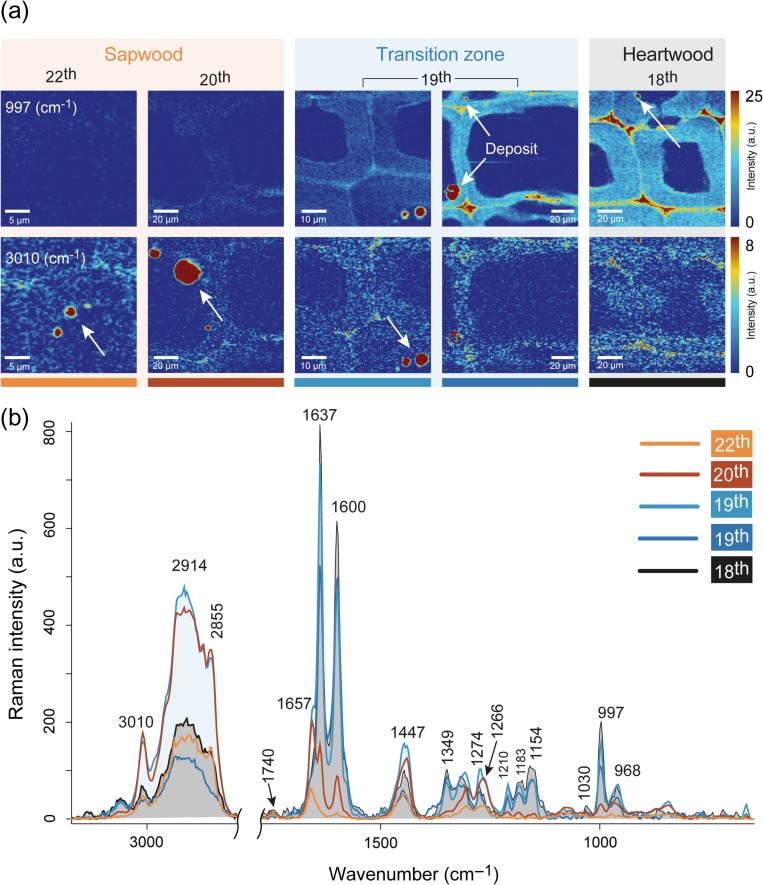
Learning from similarities and differences of various deposits. (a) Raman images including deposits in the lumen of sapwood (annual ring 22nd and 20th), transition zone (annual ring 19th) and heartwood (annual ring 18th). The first row is based on integrating the pinosylvins marker band at 997 cm^–1^, while the second row is based on the lipid band at 3010 cm^−1^ (2998–3025 cm^−1^). (b) Average spectra of the deposits showing the changing chemical composition of the deposits.

Beside in the CC and cell wall pinosylvins have been detected in deposits in the lumen (Figure 2d). By integrating the pinosylvins band 997 cm^−1^ deposits of various sizes have been found in the transition zone and in the heartwood (Figure 4a). On the contrary, the band at 3010 cm^−1^, characteristic for lipids (Czamara et al. 2015), reveals deposits within the sapwood (Figure 4a). The deposits in the lumen are always stuck to the S3 layer and are large at the border between sapwood and transition zone, and become very small and even more tightly attached to the S3 in the heartwood (Figure 4a). The extracted average spectra of the visualized droplets in each annual ring show clearly the change in composition from sapwood to heartwood (Figure 4b). The spectra of the droplets in the sapwood are dominated by bands of lipids: a pronounced CH-stretching with a sharp band at 3010 cm^−1^, a strong band at 1656 cm^−1^ (stretching of C = C), 1447 cm^−1^ (bending of CH_2_/CH_3_) and 1266 cm^−1^ (deformation of CH). Comparison with linoleic and oleic acid (see Figure S3 available as Supplementary Data at *Tree Physiology* Online) confirms that the sapwood deposit spectrum resembles the fatty acid spectra with all bands clearly present. The additional band at 1740 cm^−1^ (stretching C = O, Czamara et al. 2015) is attributed to the presence of triacylglycerols (see Figure S3 available as Supplementary Data at *Tree Physiology* Online). The main phenolic stretching vibration at 1600 cm^−1^ appears as a small band in the sapwood deposit average spectrum (Figure 4b, dark orange), hence revealing a small contribution of aromatics. Closer examination of the average spectra from 20th and 19th showed a rapid change from lipids to phenolic compounds from sapwood to heartwood (Figure 4b) with strong bands at 1637 cm^−1^ (assigned to C = C stretch of PS and PSMME), 1600 cm^−1^ and 997 cm^−1^ (assigned to the 1,3,5-substituted aromatic ring of PS and PSMME). The transition zone exhibited additional peaks at 1349 cm^−1^ (CH bending of the ring and double bound) and 1154 cm^−1^ (CH bending of the ring) that are typically for pinosylvins. The highest intensity of phenolics is observed in the heartwood deposit in the annual ring 18th, which highly resembled the pinosylvins reference spectra (see Figure S3 available as Supplementary Data at *Tree Physiology* Online). The main difference was the higher intensity of the CH stretching around 3000 cm^−1^ for the deposit in the heartwood, pointing to the presence of lipids. The absence of the band at 1620 cm^−1^ and 1359 cm^−1^ allows to conclude that the heartwood deposits share a high proportion of PSMME, as also confirmed by the typical PSMME band at 968 cm^−1^ (Figure 4b and see Figure S3 available as Supplementary Data at *Tree Physiology* Online).

Although in former studies no details on the chemistry of observed droplets could be given, Magel et al. (1994) claimed that lipophilic apolar droplets appear in the vicinity of the amyloplast at the beginning of heartwood formation. These droplets stick to the pits and further to the lumen of neighbor tracheids after the disintegration of the protoplast. Fromm (2013) concluded that the cell walls in heartwood are infiltrated with these droplets. Also, Schneider et al. (2003) found a lipidic lining through the xylem of *Myrothamnus flabellifolia* originating from rays. A recent study by Belt et al. (2017) showed filled lumen and small deposits in the middle heartwood, interpreted as resin acids and pinosylvins, respectively. In our study, we were able to show the transition of the droplets from a lipidic to an aromatic character. The deposits in the sapwood contain mostly lipids (Figure 4), but the spectra also reveal a small amount of phenolic components by the bands at 1600 cm^−1^ and 1349 cm^−1^. On the other hand, the deposits found in heartwood have still minor lipidic traces, confirming the co-existence of lipids and aromatics once again. It is known that the pinosylvins in the transition zone are synthesized from triglycerides (besides from sucrose) (Lim et al. 2016). So the fatty acids and triglycerides are involved in the synthesis, but could also play a role in transportation.

### Discrimination between lipophilic and hydrophilic part of heartwood

**Figure 5.**
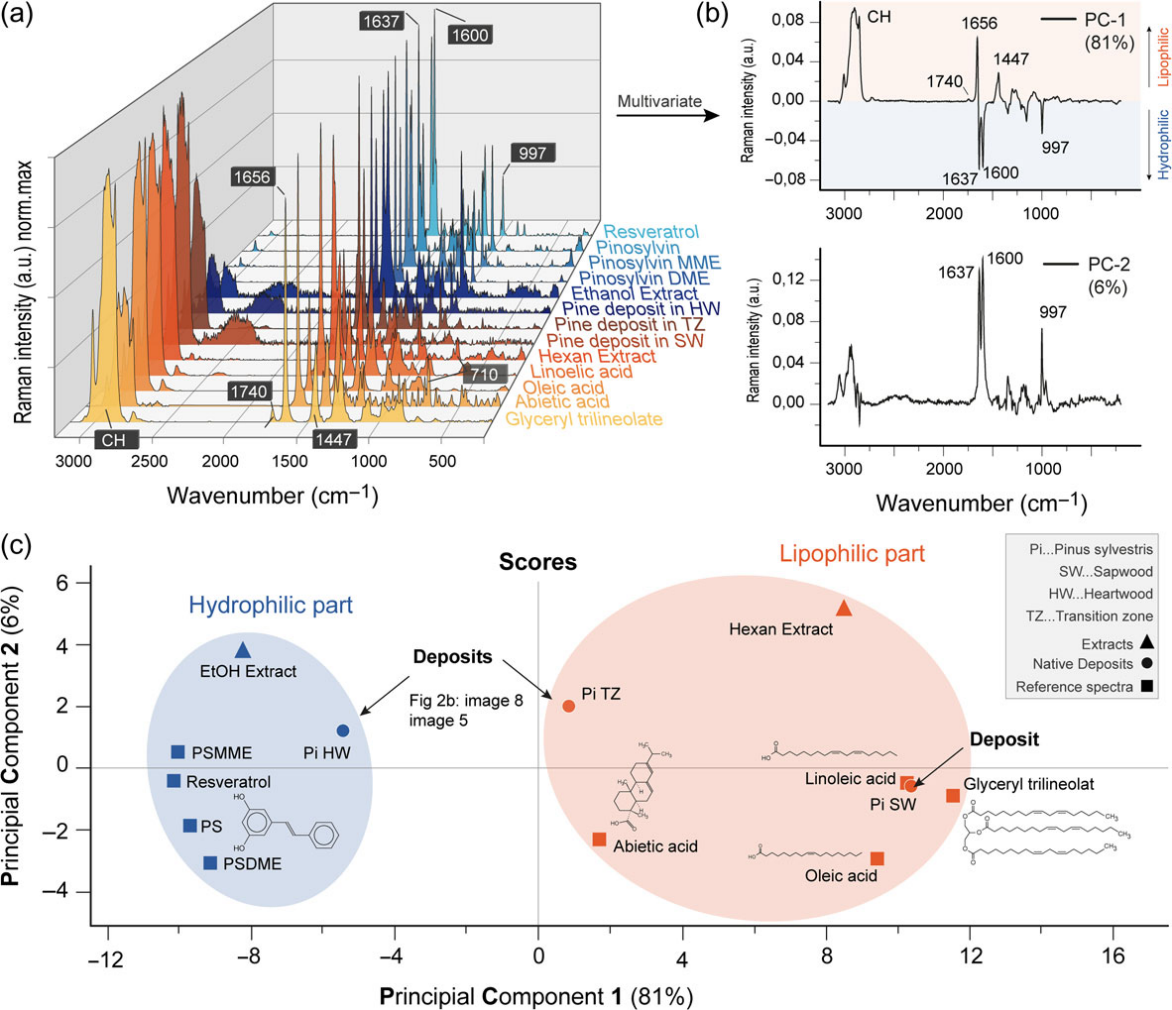
Lipophilic or hydrophilic? Multivariate comparison of spectra from pine references, extractives and native deposits. (a) Raman spectra of extractives reference compounds grouped in lipophilic (orange) and hydrophilic (blue) components compared with deposits in sapwood (SW, position 22nd), transition tone (TZ, image 5) and heartwood (HW, image 8) of native pinewood (Figure 2) as well as the Hexan and EtOH extract. The spectra are baseline corrected using concave rubberband (polynomial 6) and min–max normalized in a frequency range from 200 to 3170 cm^−1^. (b) Loadings (PC-1 and PC-2) of the PCA. Note that these are an abstract representation of the spectral profiles with negative values. PC-1 distinguishes clearly the lipophilic part (CH-stretch, 1656 and 1447 cm^−1^) with the positive values whereas the negative values represent the hydrophilic part with the stilbene marker bands 1637, 1600 and 997 cm^−1^. PC-2 shows mostly the hydrophilic part. (c) PCA scores (PC-1 vs PC-2; 87% of model explicability) separate all the spectra into a hydrophilic (blue) and lipophilic (red) group.

**Figure 6.**
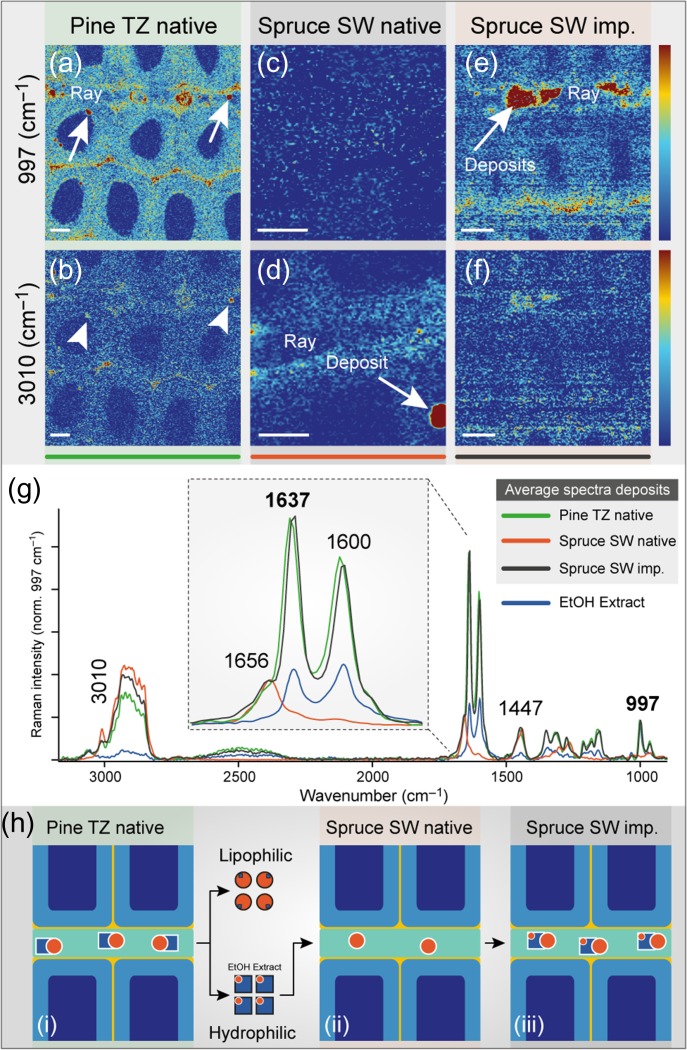
Pinosylvins transported through radial rays. (a, b) Raman images of native pine wood in the transition zone (c, d) native spruce sapwood and (e, f) spruce sapwood after impregnation with EtOH extract of pine. The first row is based on integrating the pinosylvins marker band at 997 cm^−1^, while the second row is based on the lipid band at 3010 cm^−1^ (2998–3025 cm^−1^). The intensity scale is from 0 to 5 for all images (a–f), except image (d) (0–15). (g) Comparison of the extracted average spectra from each deposit and the EtOH extract (blue spectrum). (h) Sketch of the observed results: (i) the native pinewood contains hydrophilic and lipophilic compounds collocated in deposits (see also Figures 2 and 4); (ii) these components can be extracted separately and only the hydrophilic fraction (mainly pinosylvins) be used for impregnation of native spruce sapwood; and (iii) thereby the same result as in native pine transition zone is achieved.

The extractives of pine wood can be divided into a hydrophilic (pinosylvins and lignans) and a lipophilic fraction (resin acids, fatty acids and triglycerides) (Willför et al. 2003, Nuopponen et al. 2004*a*, 2004*b*). By sequential extraction, these lipophilic and hydrophilic components of pine wood were further investigated. The acquired Raman spectra of these extracts were compared with reference compounds (Figure 5a; for a detailed comparison, see Figure S3 and the Assignment Tables S1–S8 available as Supplementary Data at *Tree Physiology* Online) and subjected to PCA (Figure 5b and c). The first principal component PC-1 explains 81 % of the total variance in the data and demonstrates significant spectral differences between lipophilic (orange) and hydrophilic (blue) components (Figure 5b and c). The loadings give the wavenumbers that are important for this group separation (Figure 5b). The major variation was found to be associated either with the bands at 1447, 1656 cm^−1^ and the CH region (corresponding to the lipophilic part) or to the bands at 997, 1600 and 1637 cm^−1^ (hydrophilic part). The PCA score plot shows clearly that the EtOH extract (Figure 5c) and the native deposit from the heartwood (Pi HW) belong to the hydrophilic part and are closely related to the PSMME. This observation is in accordance with Willför et al. (2003), who reported an average content of the pinosylvins in the mature heartwood of pine with a share of 6.76 mg g^−1^ PSMME (the highest amount), 4.20 mg g^−1^ PS and 0.07 mg g^−1^ PSDME. Within the lipophilic side, abietic acid and the deposit in the transitions zone (Pi TZ) are the ones closest to the hydrophilic part. This is due to the higher amount of stilbenes in the deposit (see spectrum Figure S3 available as Supplementary Data at *Tree Physiology* Online) and the rather low CH-stretching of the abietic acid compared with the fatty acids (Figure 5a; see Figure S3 available as Supplementary Data at *Tree Physiology* Online). The chemical structure of abietic acid is different as it belongs to the resin acids. Several authors (Hillis 1987, Willför et al. 2003, Nuopponen et al. 2004*a*, Ekeberg et al. 2006) have shown that abietic acid is the most abundant resin acid in pine heartwood. The hexane extract spectrum reflects contributions of resin acids by the band at 710 cm^−1^ (see Figure S3 available as Supplementary Data at *Tree Physiology* Online). The authors showed also that the linoleic and oleic acid are the most common fatty acids in Scots pine sapwood. Based on spectral signature of sapwood deposits, located in the score plot very near linoleic acid (Figure 5c), it is revealed that these lipophilics are mainly found within the lumen of sapwood. Furthermore, the sapwood deposits show also an additional band at 1740 cm^−1^ (stretching C = O; Czamara et al. 2015) similar to the glyceryl trilineolate (Figure 5a; see Figure S3 available as Supplementary Data at *Tree Physiology* Online), also reflected in the PC-1 loading plot (Figure 5b). Based on this band it is possible to conclude that the native deposits in the sapwood contain triglycerides. Bergström (2003) detected high amounts of triglycerides in sapwood, while free fatty acids were higher in transition zone and heartwood. These lipids are, in our study, mainly found in the deposits in the lumen (Figure 4a) and in rays (Figure 6).

### Tracking in-situ extractives in native rays and during in-vitro impregnation

Radial rays in the transition zone of pine also reveal deposits, as highlighted in Figure 6a (white arrows) by integrating the marker pinosylvin band at 997 cm^−1^. Furthermore, the integration of the band 3010 cm^−1^ indicates the presence of lipidic compounds at the same positions (Figure 6b, white arrowheads), confirming again the co-localization and close relationship between pinosylvins and lipids. Native spruce sapwood lacks pinosylvins (Figure 6c) as reported in the literature (Hovelstad et al. 2006), but lipids were present in reasonable amounts (Figure 6d). Impregnating the pinosylvin-free spruce sapwood with hydrophilic pine extract results in a similar pinosylvins distribution pattern like in pine heartwood (Figure 6e; see Figure S4a available as Supplementary Data at *Tree Physiology* Online). Within the ray, the highest pinosylvins amount was detected and associated with lipids (Figure 6f).

The average spectra from the deposits of native pine and impregnated spruce (Figure 6g) coincide very well, pointing to the same chemical composition. Although the EtOH extract used for impregnation contains only a small amount of lipids, the deposits found after impregnation show strong lipid bands. These typical high signal in the CH region around 2900 cm^−1^, the shoulder 1656 cm^−1^ and the band at 1447 cm^−1^ are present in the average spectrum of the deposit in native spruce sapwood (Figure 6g). Therefore, these native lipids seem to be involved in the transport of pinosylvins also in the artificial impregnation of spruce. We thus hypothesize that the hydrophilic pinosylvins are associated with the lipids for transportation as summarized in Figure 6h in a schematic drawing.

Transport of stilbenes with lipids is possible and was reported by Fahr et al. (2005), who described delivering of resveratrol in vesicles. Indeed, due to the low water solubility of pinosylvins (Hemingway and Laks 2012), its transport could be facilitated by lipid micelles and vesicles (Stuart and Boekema 2007, Silva et al. 2014). Besides, Barros et al. (2015) suggest for the extracellular transport of lignin monomers during lignification also a vesicle-associated exocytosis. Heartwood formation is, according to Gang et al. (1998), a kind of a ‘second lignification’ and this study proves indeed similar accumulation sites and transportation mechanisms for aromatic extractives. Besides the importance of lipids for transportation, it is suggested that they seal pits to block the water transport and in this way selectively deactivate parts of its water conduction system (Schneider et al. 2003).

## Conclusion

Even though the heartwood formation occurs on the macroscale within one annual ring in pine, Raman microscopy enabled us to visualize the components involved in heartwood formation on the microscale. In particular, the Raman spectral characteristics allowed us to capture the different cellular components (e.g., cell wall layers, CML and pits) in addition to its single constituents (i.e., lignin, cellulose, pinosylvins, fatty acids and triglycerides) at the different sub-cellular layers. In particular, the ultrastructural mapping of cellular compartments and the comparison with Raman reference spectra at these positions allowed us to find a symbiotic relationship between pinosylvins and lipids. We observed a higher amount of pinosylvins in the CC, CML, pits and the lumen sided S3 layer. However, we found that lipids are located preferably in different deposits in radial rays and the lumen together with pinosylvins. We hypothesize that this relationship is essential for the synthesis and transport of pinosylvins. Furthermore, these results show the importance of the distribution on the micron-level of extractives and how their location is optimized to block the water and fungal hyphae pathways.

## Supplementary Material

Supplementary Data

Supplementary Data
