# Supplementary material for: Antifungal stilbene impregnation: transport and distribution on the micron-level
Source: Tree Physiol. 2018 Jul 10;38(10):1526–37. doi: 10.1093/treephys/tpy073 (PMC6198867; doi:10.1093/treephys/tpy073)
Supplement: Supplementary Data [file tpy073_reference_spectra.pdf]

Pinosylvin

European Research Council
Established by the European Commission
SCATAPNUT
[681885]

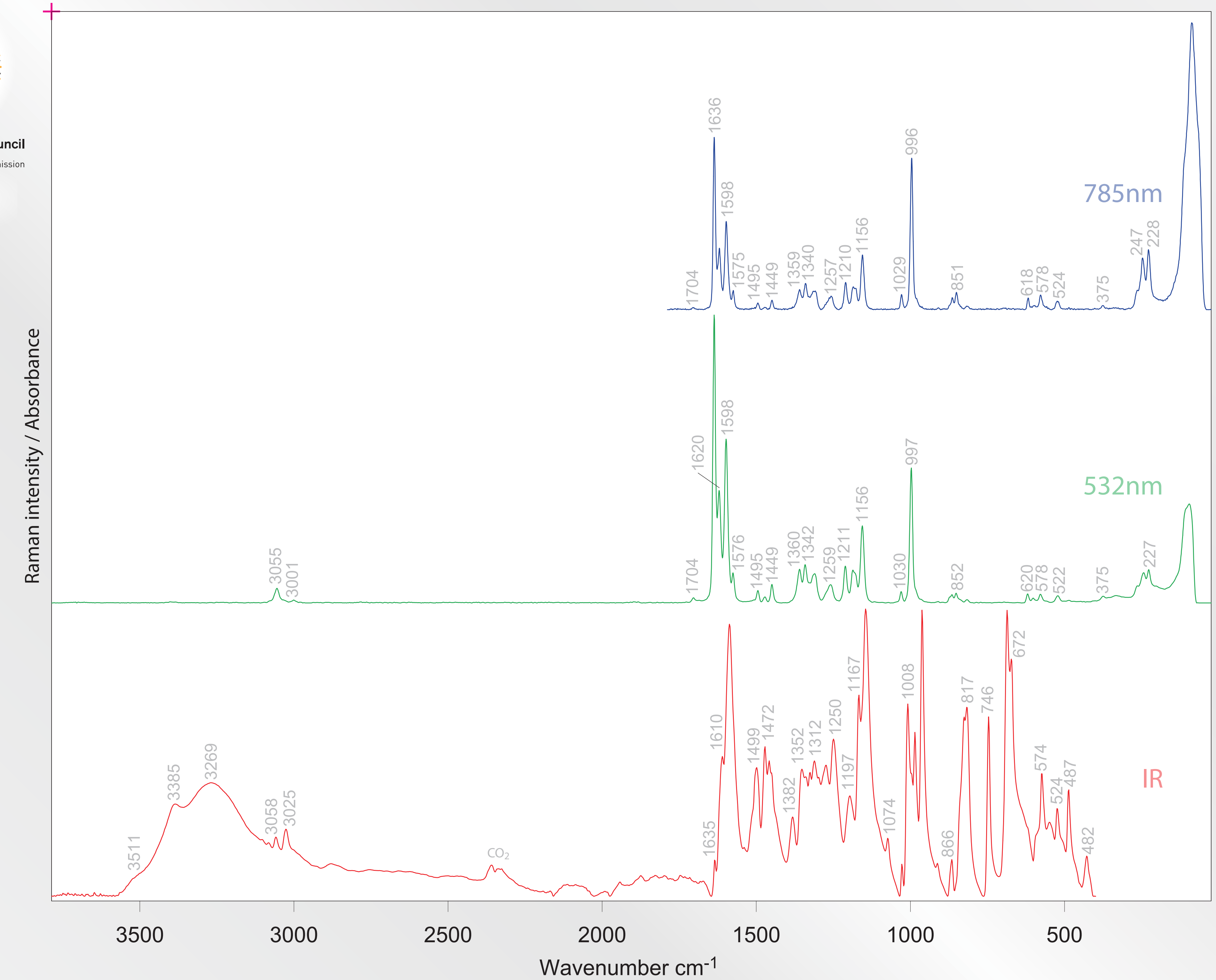

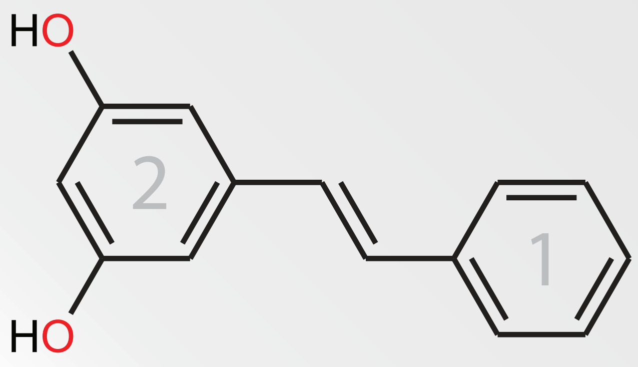

Sample:	56297	Mode:	Vertex ATR
Producer:	Sigma	n_{Acc}:	32
Purity:	97%		
State:	crystalline		
Temperature:	n.d./21.3°C		
measured in:	neat/H_2O		

λ_{ex}:	532.083 nm	λ_{ex}:	784.940 nm
LPO:	15 mW	LPO:	81,4 mW
ν (Si):	520.03 cm^{-1}	ν (Si):	520.07 cm^{-1}
t_{int}:	10x0.14756 s	t_{int}:	0.04372 s
Grating:	600 g/mm	Grating:	600 g/mm
p_{Laser}:	0°	p_{Laser}:	0°
$p_{Spectrometer}$:	unpol	$p_{Spectrometer}$:	unpol
Objective:	20x air NA 0.4	Objective:	20x air NA 0.4

IR	532	785	*R1*	*R2*	
3511					ν O-H
3385					ν O-H
3269					ν O-H
3103					ν C-H of ring
3082			***2***		ν C-H of R1
3058	3055				ν C-H of ring
3025					ν C-H of ring/C=C
	3001				ν C-H of ring/C=C
2879					
	1704	1704			
1635	***1636***	***1636***			ν C=C
	1620	1620		***8a***	ν C=C of R2
1610				***8a***	ν C=C of R2
	1598	***1598***	***8a***		ν C=C of R1
1587				***8b***	ν C=C of R2
	1576	1575	***8b***		ν C=C of R1
1515				***19a***	ν C=C of R2
1499	1495	1495	***19a***		ν C=C of R1
1472	1472	1472	*19b*	***19b***	ν_{ip} [C=C of ring]
1458			***19b***	*19b*	ν C=C of ring
1451	1449	1449	*19b*	*19b*	ν_{op} [C=C of ring]
1382			*3*	*14*	δ C-H
1352	1360	1359	*3*	***14***	ν C=C of R2; δ C-H; δ O-H
1340	1342	1340	*3*	***13***	ν_{ip} [CΦ-O, CΦ-C]; δ C=C of R2; δ C-H
1326					δ C-H
1312	1311	1309			δ C-H
1298			*14*	***3***	δ C-H of ring R2
1274			*14*	*14*	δ C-H
	1259	1257			
1250	1211	1210	***13***	*13*	ν CΦ-C; δ C=C of ring R1
1197					
	1187	1185	***9a***	***18a***	δ O-H, δ C-H of ring
	1178	1178	***9a***	***18a***	δ O-H, δ C-H of ring
1167				***7a***	ν_{op} [CΦ-O, CΦ-C]; δ C=C of R2
	1156	1156		***7a***	ν_{op} [CΦ-O, CΦ-C]; δ C=C of R2
1145				***7b***	ν_{op} [CΦ-O]; δ C=C of R2; δ O-H
1102					
1074			***18b***		δ C-H of ring
1027	1030	1029	***18a***		δ C-H of ring
1008					
997	***997***	***996***	***12***	***12***	δ C=C of ring
985				***7b***	ν_{op} [CΦ-O, CΦ-C]; δ C=C of R2
962					γ_w C-H of C=C
913		909	***10b***		γ_t C-H of C=C; γ C-H of R1
866	865	864	***1***		ν C=C of R1
	852	851	***10a***		γ C-H of R1
838				***17a***	γ C-H of R2
826				***11***	γ C-H of R2
817	817	818		***11***	γ C-H of R2
746			***11***		γ C-H of R1
686			***4***		γ C=C of R1
672				***4***	γ C=C of R2
621	620	618	***6b***		δ C=C of R1
	602	597	***6a***		δ C=C of R1
590					
574	578	578	***6a***	***1***	ν/δ C=C of ring
549			*6a*	***6a***	δ C=C of ring
524	522	524	*6b*	***6b***	ν/δ C=C of ring
487			***16b***		γ C=C of R1
428					
	375	375			
	333				
	264				
	243	247			
	227	228			
	95				

ν...stretch, δ...in-plane bend, γ...out-of-plane bend, τ...torsion, γ_r...rocking, γ_w...wagging, γ_t...twisting Φ...ring (Varsanyi), d...degenerate s...symmetric/scissoring, as... a(nti-)symmetric, ip...in-phase, op...out-of-phase

Peter Bock, 21.03.2018

European Research Council
Established by the European Commission
SCATAPNUT
[681885]

Pinosylvinmonomethylether

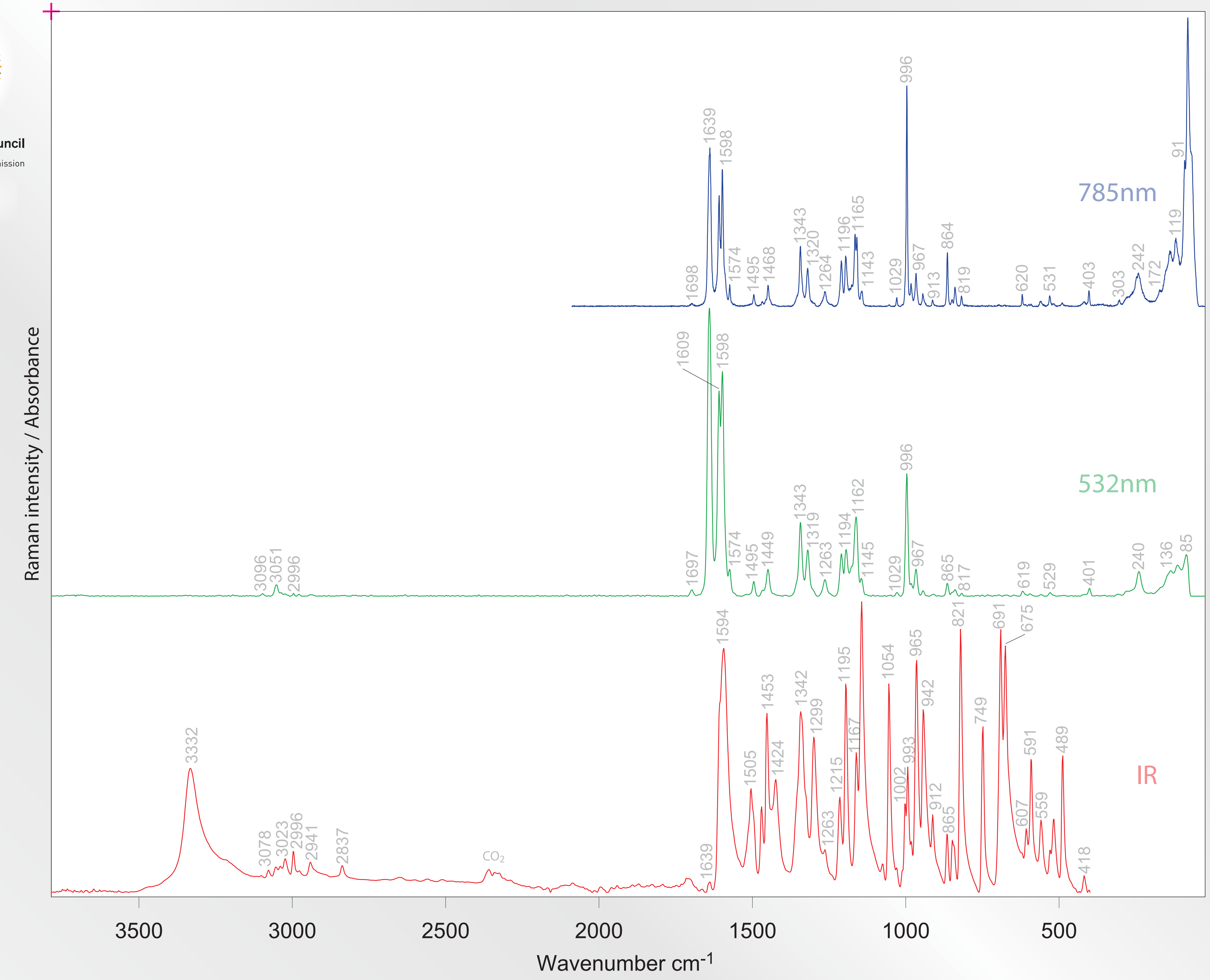

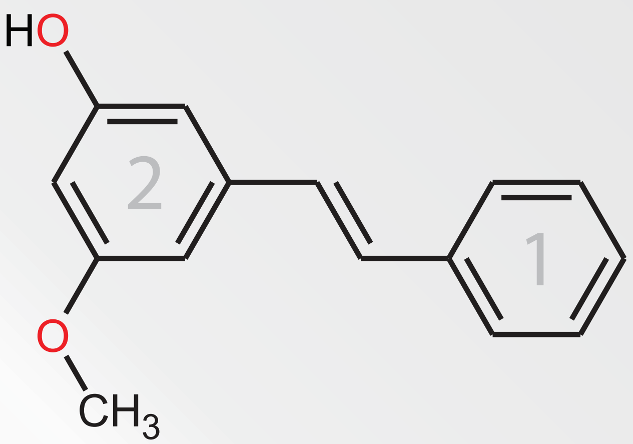

Sample:	42914	Mode:	Vertex ATR
Producer:	Sigma	n_{Acc}:	32
Purity:	97%		
State:	crystalline		
Temperature:	21.3°C		
measured in:	-		

λ_{ex}:	532.001 nm	λ_{ex}:	784.940 nm
LPO:	10.1 mW	LPO:	79.10 mW
ν (Si):	518.62 cm^{-1}	ν (Si):	520.07 cm^{-1}
t_{int}:	1.09765 s	t_{int}:	0.13372 s
Grating:	600 g/mm	Grating:	1200 g/mm
p_{Laser}:	0°	p_{Laser}:	0°
$p_{Spectrometer}$:	unpol	$p_{Spectrometer}$:	unpol
Objective:	20x air NA 0.4	Objective:	20x air NA 0.4

IR	532	785	*R1*	*R2*	
3332					ν O-H
	3096				ν C-H of ring
3078					ν C-H of R1
3053	3051				ν C-H of ring
3039	3035				ν C-H of ring
3023	3023				ν C-H of C=C
2996	2996				ν_{as} C-H of CH_3
	2979				ν_{as} C-H of CH_3
2941	2943				ν_{as} C-H of CH_3
2837					ν_s C-H of CH_3
1710	1697	1698			
1639	***1640***	***1639***			ν C=C
1606	***1609***	***1608***		***8a***	ν C=C of R2
1594	***1598***	***1598***	***8a***		ν C=C of R1
	1574	1574	***8b***		ν C=C of R1
1505	1495	1495	***19a***	***19a***	ν C=C of ring
1470		1468			δ_{as} C-H of CH_3
1453	1449	1449	***19b***		ν C=C of R1
1424				***19b***	ν C=C of R2
1342	1343	1343	***3***	***14***	δ C-H of R1 and C=C
	1319	1320		***13***	ν_{ip} [C_Φ-O, C_Φ-C]; δ C=C of R2
1299			***14***	***3***	δ C-H of R2 and C=C; ν C=C of R1
1263	1263	1264	***14***	***3***	δ C-H of R2 and C=C; ν C=C of R1
1215	1210	1210	***13***		ν C_Φ-C; δ C=C of R1
1195	1194	1196			γ_r C-H of CH_3; δ O-H; ν C_Φ-O
	1179		***9a***		δ C-H of R1
1161	1162	1165		***7a***	ν_{op} [C_Φ-O, C_Φ-C]; δ C=C of R2
		1159		***7a***	ν_{op} [C_Φ-O, C_Φ-C]; δ C=C of R2
1144	1145	1143		***7b***	ν_{op} [C_Φ-O, C_Φ-C]; δ C=C of R2
1075			***18b***		δ C-H of R1
1054		1053	*18b*	*18b*	ν O-CH_3
1030	1029	1029	***18a***		δ C-H of R1
1002	***996***	***996***	***12***		δ C=C of R1
993	***996***	***996***		***12***	δ C=C of R2
982		983	***5***		γ C-H of R1
965	967	967			γ_w C-H of C=C
942	944	944			ν_s C-O-C
912	909	913	***10b***		γ_t C-H of C=C; γ C-H of R1
865	865	864	***1***		ν C=C of R1
848		849	*10b*	***17b***	γ_t C-H of C=C; γ C-H of ring
843	839	840	***10a***		γ C-H of R1
821	817	819		***11***	γ C-H of R2
749			***11***		γ C-H of R1
691		697	***4***		γ C=C of R1
675				***4***	γ C=C of R2
	619	620	***6b***		δ C=C of R1
607					
591		591	***6a***	***1***	
559		559	***6a***	***1***	
529	529	531		***6b***	δ C=C of R2
518				***6a***	δ C=C of R2
489		491	***16b***		γ C=C of R1
418		418			δ C-O-C
	401	403	***16a***		γ C=C of R1
		303			
	240	242			
		172			
	136	139			
	114	119			
		91			
	85	81			

ν...stretch, δ...in-plane bend, γ...out-of-plane bend, τ...torsion, γ_r...rocking,
γ_w...wagging, γ_t...twisting Φ...ring (Varsanyi), d...degenerate
s...symmetric/scissoring, as... a(nti-)symmetric, ip...in-phase, op...out-of-phase

Peter Bock, 24.03.2018

Pinosylvindimethylether

European Research Council
Established by the European Commission
SCATAPNUT
[681885]

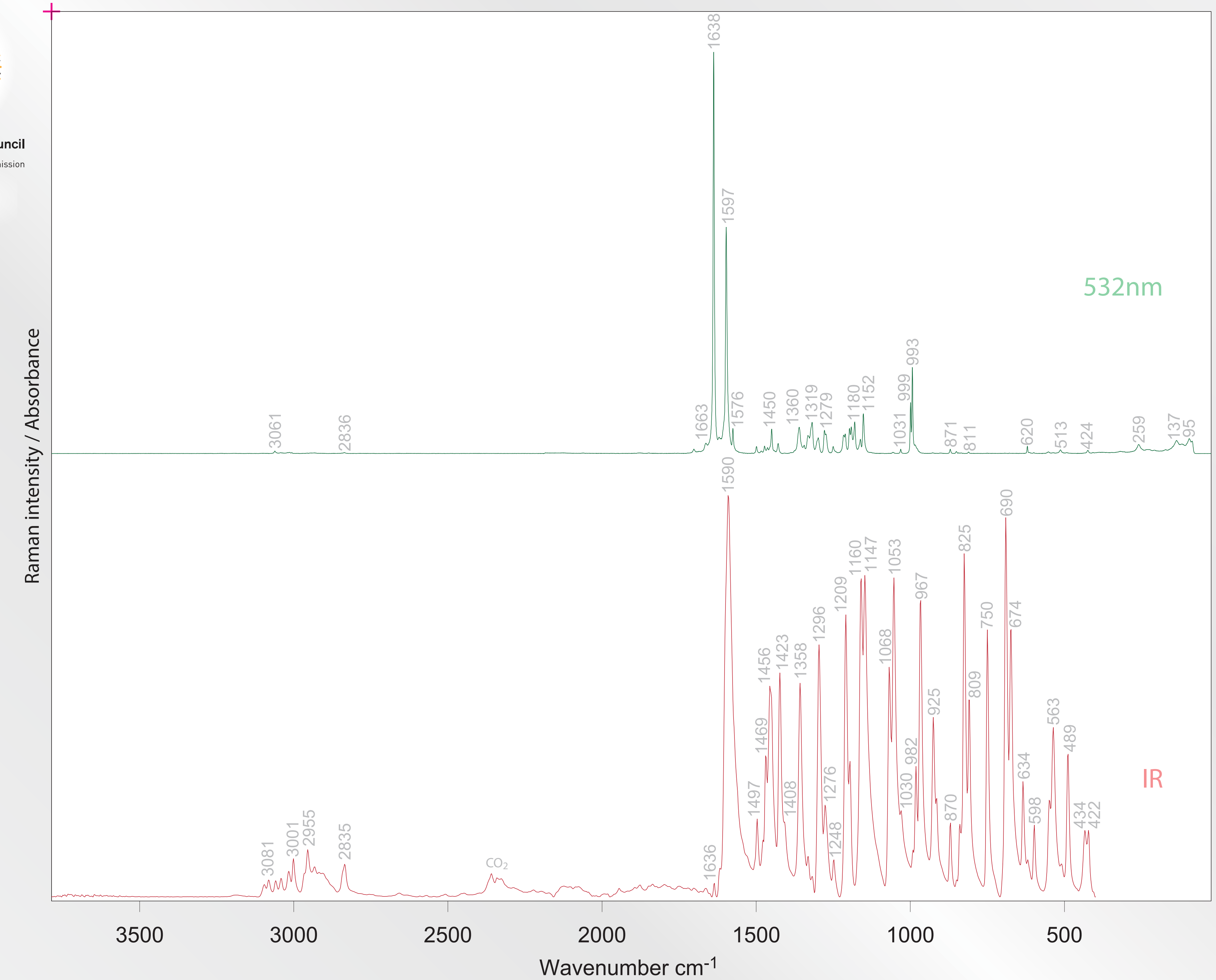

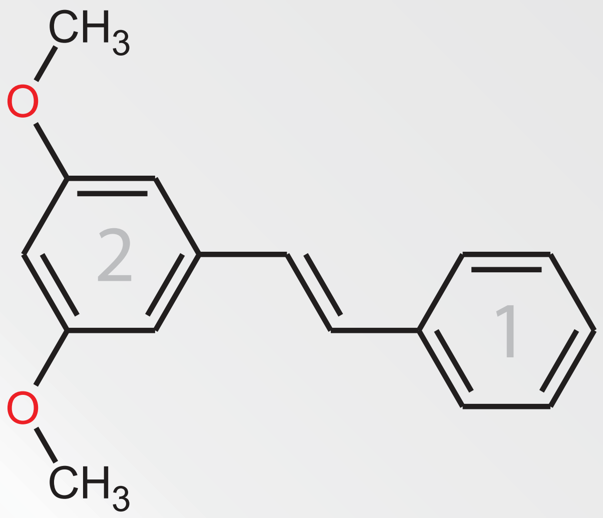

Sample:	ODS011444
Producer:	Sigma
Purity:	-
State:	crystalline
Temperature:	21°C
measured in:	neat

Mode:	Vertex ATR
n_{Acc}:	32

λ_{ex}:	532.009 nm
LPO:	31 mW
ν (Si):	519.89 cm^{-1}
t_{int}:	0.13371 s
Grating:	1800 g/mm
p_{Laser}:	0°
$p_{Spectrometer}$:	unpol
Objective:	20x air NA 0.4

IR	532	*R1*	*R2*	
3095				ν C-H of ring
3081				ν C-H of ring
3059	3061			ν C-H of R1
3041				ν C-H of ring
3016	3014			ν C-H of C=C
3001				ν_{as} C-H of CH_3
2955				ν_{as} C-H of CH_3
2932				ν_{as} C-H of CH_3
2918				ν_{as} C-H of CH_3
2835	2836			ν_{s} C-H of CH_3
	1702			
	1663			
1636	***1638***			ν C=C
1618	1621			ν C=C of ring (impurity)
1598	***1597***	***8a***	***8a***	ν C=C of ring
1590			***8b***	ν C=C of R2
	1576	***8b***		ν C=C of R1
1497	1499	***19a***		ν C=C of ring
1478	1483			
1469	1473		***19a***	
	1463			δ_{as} C-H
1456	1450	***19b***		ν C=C of R1
1423	1429		***19b***	ν C=C of R2
1408				
1358	1360	*3*	***14***	ν C=C of R2; δ C-H of R1 and C=C
	1344			δ C-H
1332	1332	***3***		δ C-H of C=C; δ C-H of R1
1318	1322	***3***		δ C-H of C=C; δ C-H of R1
	1319	***3***		δ C-H of C=C; δ C-H of R1
1296	1299		***13***	ν_{ip} [C_{Φ}-O, C_{Φ}-C], δ C=C of R2
1276	1279	*14*	***3***	δ C-H of R2 and C=C
	1274	*14*	***3***	δ C-H of R2 and C=C
1248	1250	*14*	***3***	δ C-H of R2 and C=C
	1216	***13***		ν C_{Φ}-C; δ C=C of R2
1209	1211	***13***		ν C_{Φ}-C; δ C=C of R2
1196	1197			$\gamma_{ip,r}$ [C-H of CH_3]
	1192			$\gamma_{op,r}$ [C-H of CH_3]
	1180	***9a***		δ C-H of R1
1160	1162		*7a*	$\gamma_{op,r}$ [C-H of CH_3]; δ C-H of R2; ν_{op} [C_{Φ}-O]; δ C=C of R2
	1152		***7a***	ν_{op} [C_{Φ}-O]; δ C=C of R2; $\gamma_{op,r}$ [C-H of CH_3]; δ C-H of R2
1147			***7b***	ν_{op} [C_{Φ}-O]; $\gamma_{op,r}$ [C-H of CH_3]; δ C-H of R2
1068				ν_{op} [O-CH_3]
1053	1057			ν_{ip} [O-CH_3]
1030	1031	***18a***		δ C-H of R1
	999	***12***		δ C=C of R1
991	***993***		***12***	δ C=C of R2
982	984	***5***		γ C-H of ring
967				γ_{w} C-H of C=C
925				$\nu_{ip,s}$ [C-O-C]
915				$\nu_{ip,s}$ [C-O-C]
870	871	***1***		ν C=C of R1
849	851	***10b***		γ C-H of R1
839			***10a***	γ C-H of R2
825			***11***	γ C-H of R2
809	811		***11***	γ C-H of R2
750		***11***		γ C-H of R1
690		***4***		γ C=C of R1
674				
634			***4***	γ C=C of R2
619	620	***6b***		δ C=C of R1
598				
549	552	***6a***	***6a***	δ_{ip} [C=C of ring]
536		***6a***	***6a***	δ_{op} [C=C of ring]
510	513		***6b***	δ C=C of R2
489		***16b***		γ C=C of R1
434				
422	424			
406				
	319			
	259			
	137			
	95			
	86			

ν...stretch, δ...in-plane bend, γ...out-of-plane bend, τ...torsion, γ_r...rocking, γ_w...wagging, γ_t...twisting Φ...ring (Varsanyi), d...degenerate s...symmetric/scissoring, as... a(nti-)symmetric, ip...in-phase, op...out-of-phase

Peter Bock, 23.03.2018

European Research Council
Established by the European Commission

SCATAPNUT
[681885]

Resveratrol

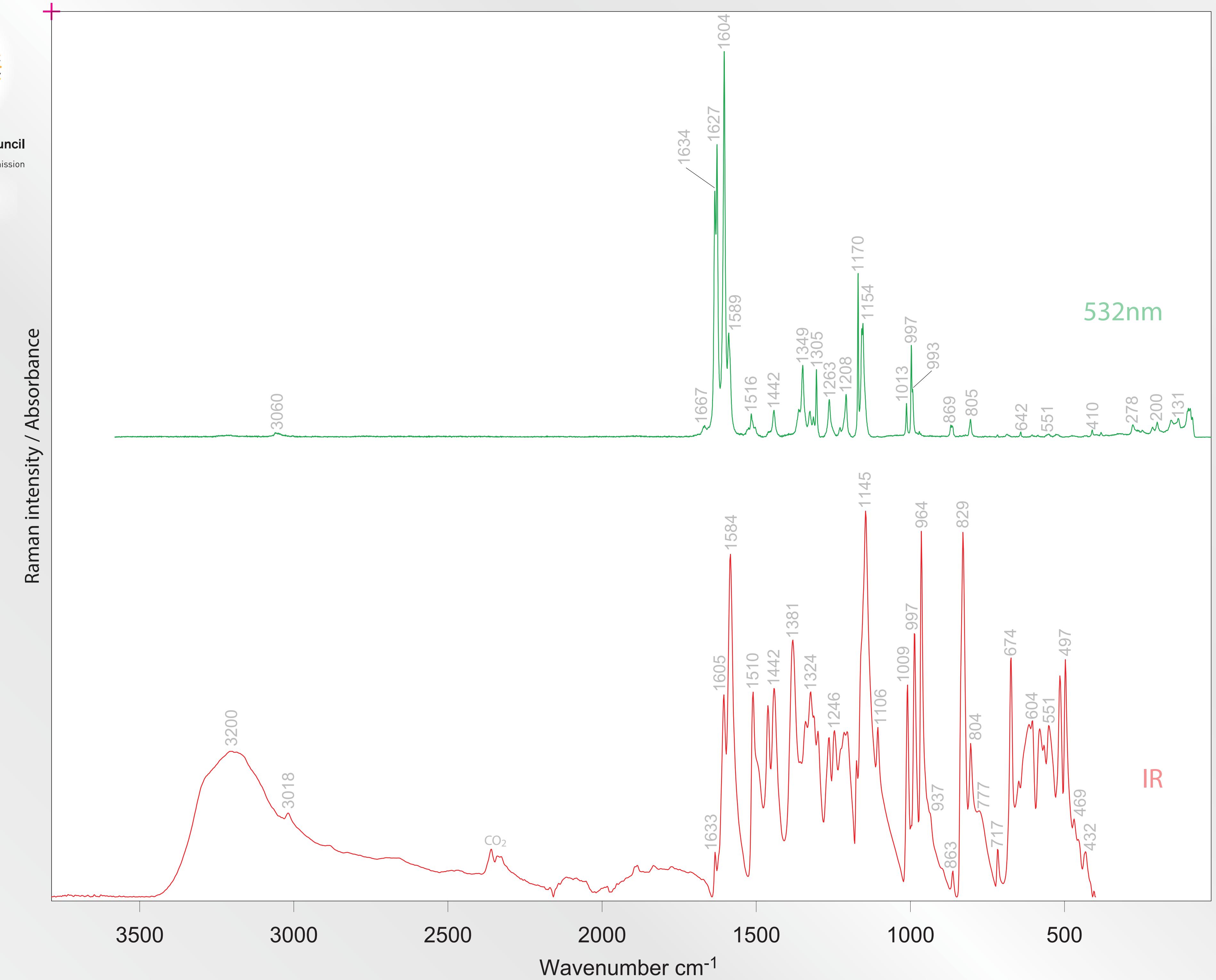

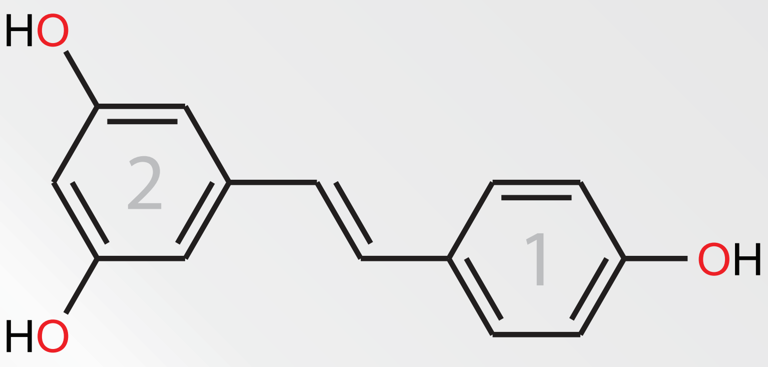

Sample:	ODS011444
Producer:	Sigma
Purity:	-
State:	crystalline
Temperature:	21°C
measured in:	neat

Mode:	Vertex ATR
n_{Acc}:	32

λ_{ex}:	532.009 nm
LPO:	31 mW
ν (Si):	519.89 cm^{-1}
t_{int}:	0.13371 s
Grating:	1800 g/mm
p_{Laser}:	0°
$p_{Spectrometer}$:	unpol
Objective:	20x air NA 0.4

IR	532
3200	3218
	3060
3018	
	1667
1633	***1634***
	1627
1605	***1604***
1584	1589
1510	1516
1495	1502
1462	1460
1444	1442
1381	
1357	1362
	1349
1340	
1324	1325
1313	1315
1300	1305
1264	1263
1246	
	1228
1215	
1205	1208
1174	***1170***
1157	***1158***
	1154
1145	
1106	
1009	1013
997	***997***
	993
987	
964	971
937	
896	
	869
863	865
829	
804	805
777	
717	717
	687
674	
648	642
615	
604	605
581	
567	
551	551
	528
515	
497	
	476
469	
456	
432	
	410
404	
	381
	278
	248
	214
	200
	154
	131
	98
	92
	85

ν...stretch, δ...in-plane bend, γ...out-of-plane bend, τ...torsion, γ_r...rocking,
γ_w...wagging, γ_t...twisting Φ...ring (Varsanyi), d...degenerate
s...symmetric/scissoring, as... a(nti-)symmetric, ip...in-phase, op...out-of-phase

Peter Bock, 28.02.2018

European Research Council
Established by the European Commission
SCATAPNUT
[681885]

Abietic acid

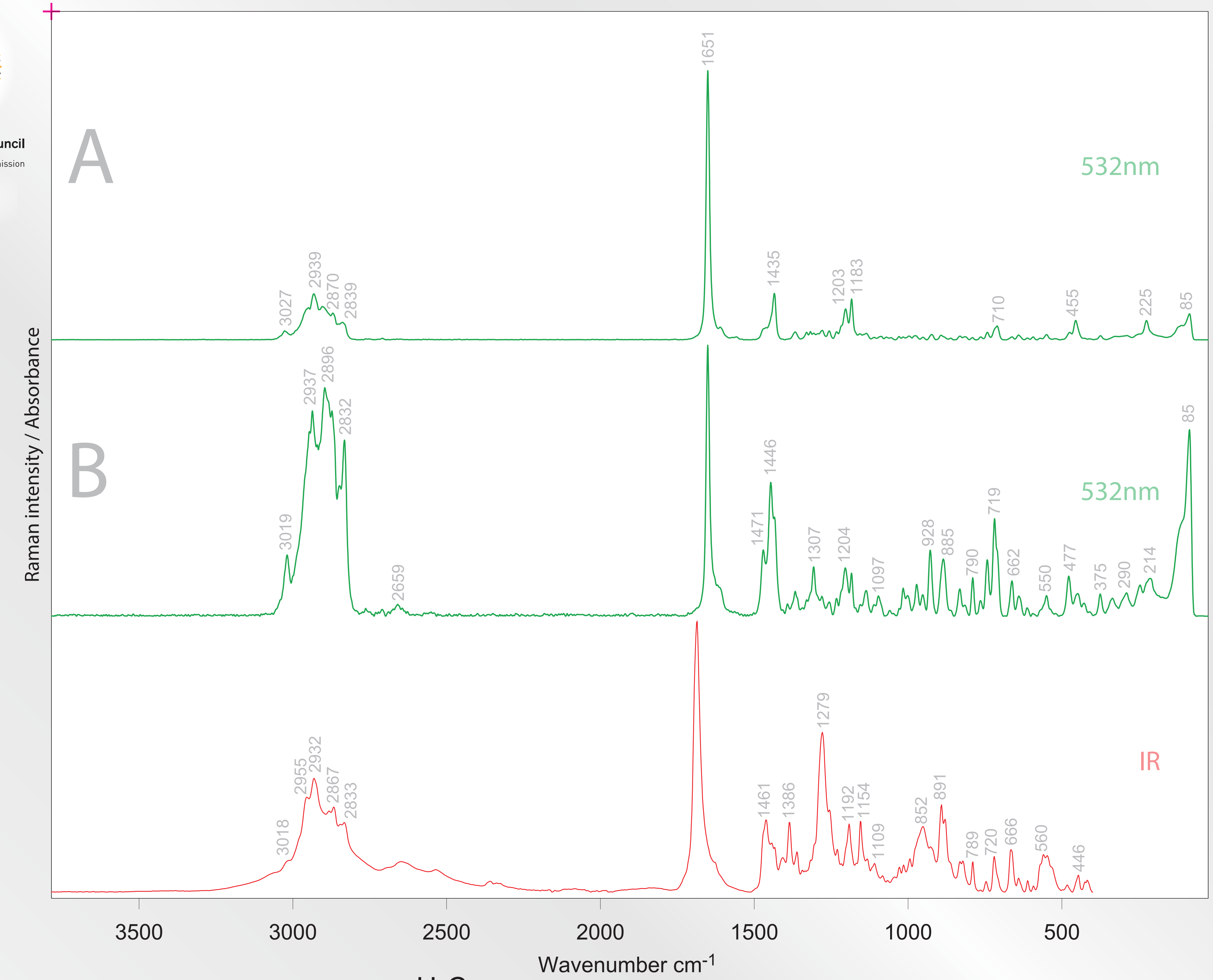

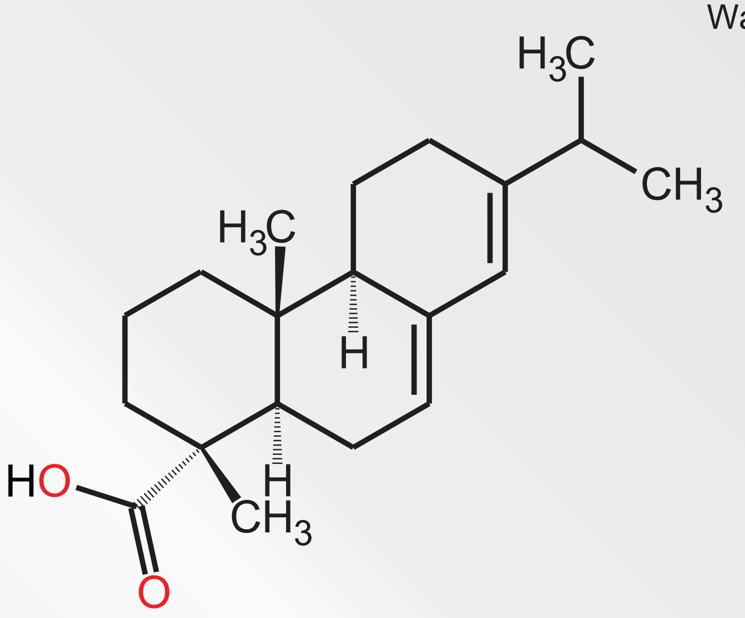

Sample: 00010
Producer: Sigma
Purity: 75%
State: crystalline
Temperature: 23°C
measured in: neat

Mode: Vertex ATR
n_{Acc}: 32

λ_{ex}: 532.001 nm
LPO: 30.1 mW
ν (Si): 520.59 cm^{-1}
t_{int}: 0.04371 s
Grating: 600 g/mm
p_{Laser}: 0/90°
$p_{Spectrometer}$: unpol
Objective: 20x air NA 0.4

IR	532 A	532 B
3018	3027	3019
2955	2949	***2946***
2932	2932	***2937***
		2921
	2904	***2896***
2883		
2867	2870	***2873***
	2853	***2848***
2833	2839	***2832***
	2763	2763
	2752	
	2709	2710
2648		2659
2536		2565
1686		
	1651	***1651***
	1611	1619
	1571	1575
	1558	1564
		1502
		1471
1461	1461	***1446***
1444		
1434	1435	1434
1408		
1386		1392
1361	1367	1367
1344		1343
1371		
	1330	1330
	1317	
1305	1306	1307
	1293	
1279	1279	1280
1255	1256	1256
1230	1232	1233
1192	1203	1204
	1183	1184
1154	1157	1155
1133	1136	1136
1109	1111	1111
1083	1087	1096
1066	1071	
1058	1056	1060
	1029	1029
1014	1016	1016
994	997	1001
	975	972
952	951	952
927	923	928
891	893	885
880		
862	861	
831	831	832
822	814	816
789	790	790
	764	764
746	742	742
720		***719***
	710	
666	662	662
640	641	640
611	612	613
592	593	
570	570	
560		
547	550	550
	522	
482		478
	474	
	455	
446		448
	428	428
417	411	
	375	375
	327	335
	304	
	288	289
	249	246
	225	
		213
	111	112
	85	85

ν...stretch, δ...in-plane bend, γ...out-of-plane bend, τ...torsion, $γ_r$...rocking, $γ_w$...wagging, $γ_t$...twisting Φ...ring (Varsanyi), d...degenerate
s...symmetric/scissoring, as... a(nti-)symmetric, ip...in-phase, op...out-of-phase

Peter Bock, 16.05.2018

European Research Council
Established by the European Commission
SCATAPNUT
[681885]

Oleic acid

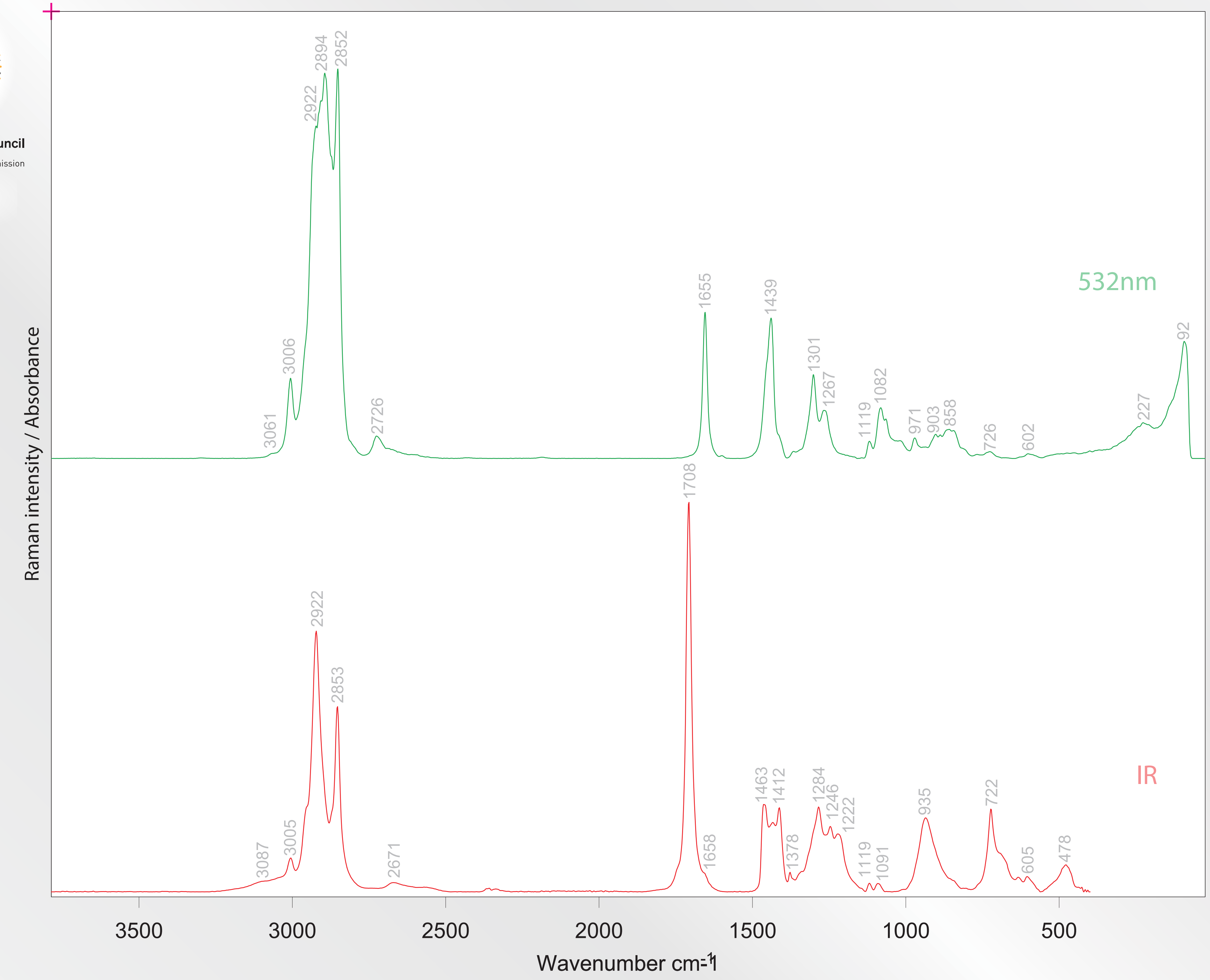

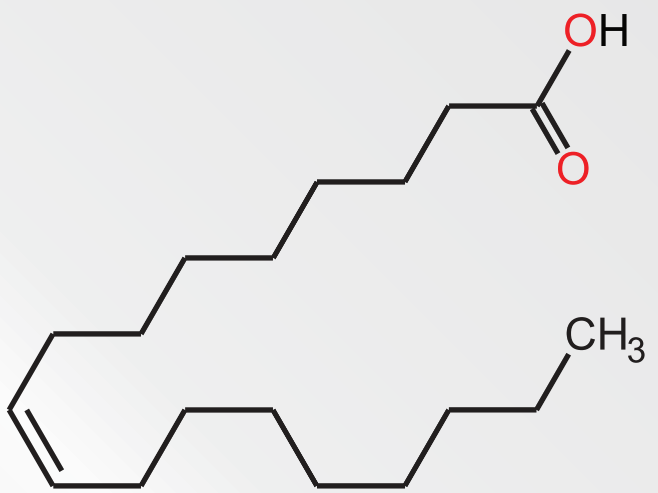

Sample:	-	Mode:	Vertex ATR
Producer:	Sigma	n_{Acc}:	32
Purity:	-		
State:	liquid		
Temperature:	21°C		
measured in:	neat		

λ_{ex}:	532.001 nm
LPO:	39.6 mW
ν (Si):	-
t_{int}:	5.09756 s
Grating:	600 g/mm
p_{Laser}:	0°
$p_{Spectrometer}$:	unpol
Objective:	20x air NA 0.4

IR	532	
3087		
3005	3006	ν C-H of C=C
2954		ν_{as} C-H of CH_3
2922	***2922***	ν_{as} C-H of CH_2
	2894	ν_{as} C-H of CH_2
2853	***2852***	ν_{s} C-H of CH_2
	2726	$2\delta_{as}$ C-H of CH_2 (overtone)
2671		
1708		ν C=O
1658	1655	ν C=C
1463		δ_{s} C-H of CH_2
1434	1439	δ_{s} C-H of CH_2
1412		δ_{s} C-H of CH_2
1378		δ_{s} C-H of CH_3
1339		δ C-H
	1301	γ_{t} C-H of CH_2
1284		$\gamma_{w/t}$ C-H of CH_2
	1267	$\gamma_{w/t}$ C-H of CH_2
1246		$\gamma_{w/t}$ C-H of CH_2
1222		$\gamma_{w/t/r}$ C-H of CH_2
1119	1119	$\gamma_{w/t/r}$ C-H of CH_2
1091		$\gamma_{w/t/r}$ C-H of CH_2
	1082	
	1065	
	1016	
	971	
935		γ_{w} C-H of C=C
	903	
	888	
	858	
	843	
	810	
	767	
722		γ_{r} C-H of CH_2
634	629	
605	602	
478		
	227	
	92	

ν...stretch, δ...in-plane bend, γ...out-of-plane bend, τ...torsion, γ_r...rocking, γ_w...wagging, γ_t...twisting Φ...ring (Varsanyi), d...degenerate s...symmetric/scissoring, as... a(nti-)symmetric, ip...in-phase, op...out-of-phase

Peter Bock, 24.03.2018

European Research Council

Established by the European Commission

SCATAPNUT

[681885]

Linoleic acid

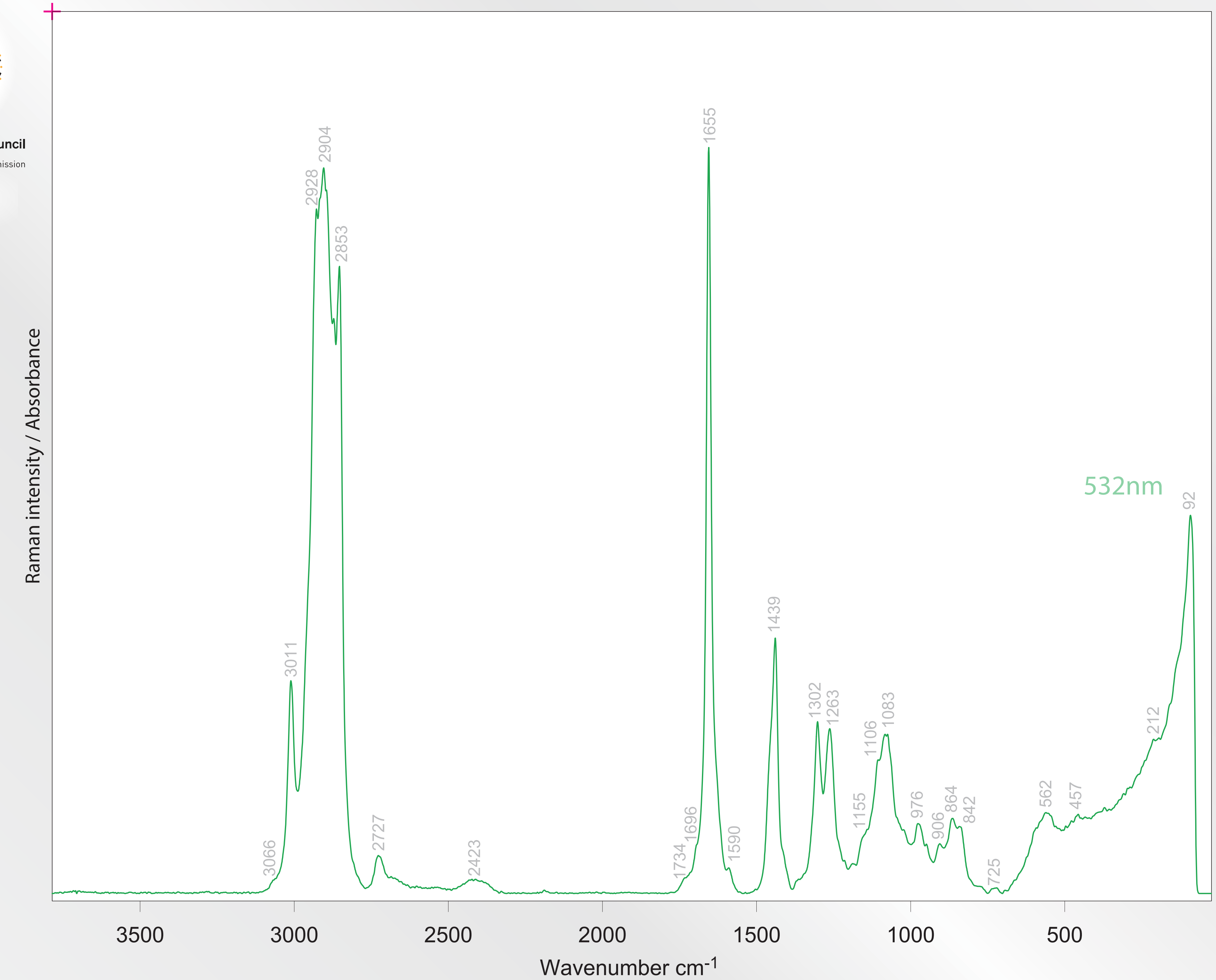

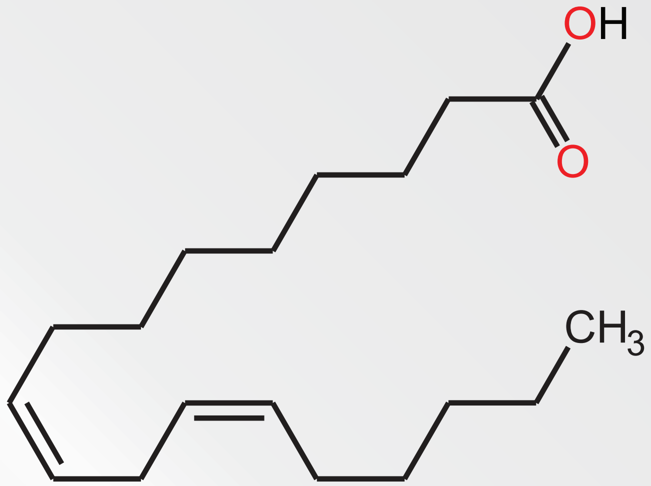

Sample:	L1376
Producer:	Sigma
Purity:	99%
State:	liquid
Temperature:	21°C
measured in:	neat

Mode:	Vertex ATR
n_{Acc}:	32

λ_{ex}:	532.001 nm
LPO:	39.6 mW
ν (Si):	-
t_{int}:	5.09726 s
Grating:	600 g/mm
p_{Laser}:	0°
$p_{Spectrometer}$:	unpol
Objective:	20x air NA 0.4

532	
3066	.
3011	
2928	
2904	
2896	
2872	
2853	
2727	
2423	
1734	
1696	
1655	
1590	
1439	
1364	
1302	
1263	
1215	
1188	
1155	
1106	
1083	
1075	
1023	
976	
949	
906	
864	
842	
776	
725	
562	
457	
212	
92	

ν...stretch, δ...in-plane bend, γ...out-of-plane bend, τ...torsion, γ_r...rocking, γ_w...wagging, γ_t...twisting Φ...ring (Varsanyi), d...degenerate s...symmetric/scissoring, as... a(nti-)symmetric, ip...in-phase, op...out-of-phase

Peter Bock, 26.03.2018

European Research Council
Established by the European Commission

SCATAPNUT
[681885]

Glyceryl trilineolate

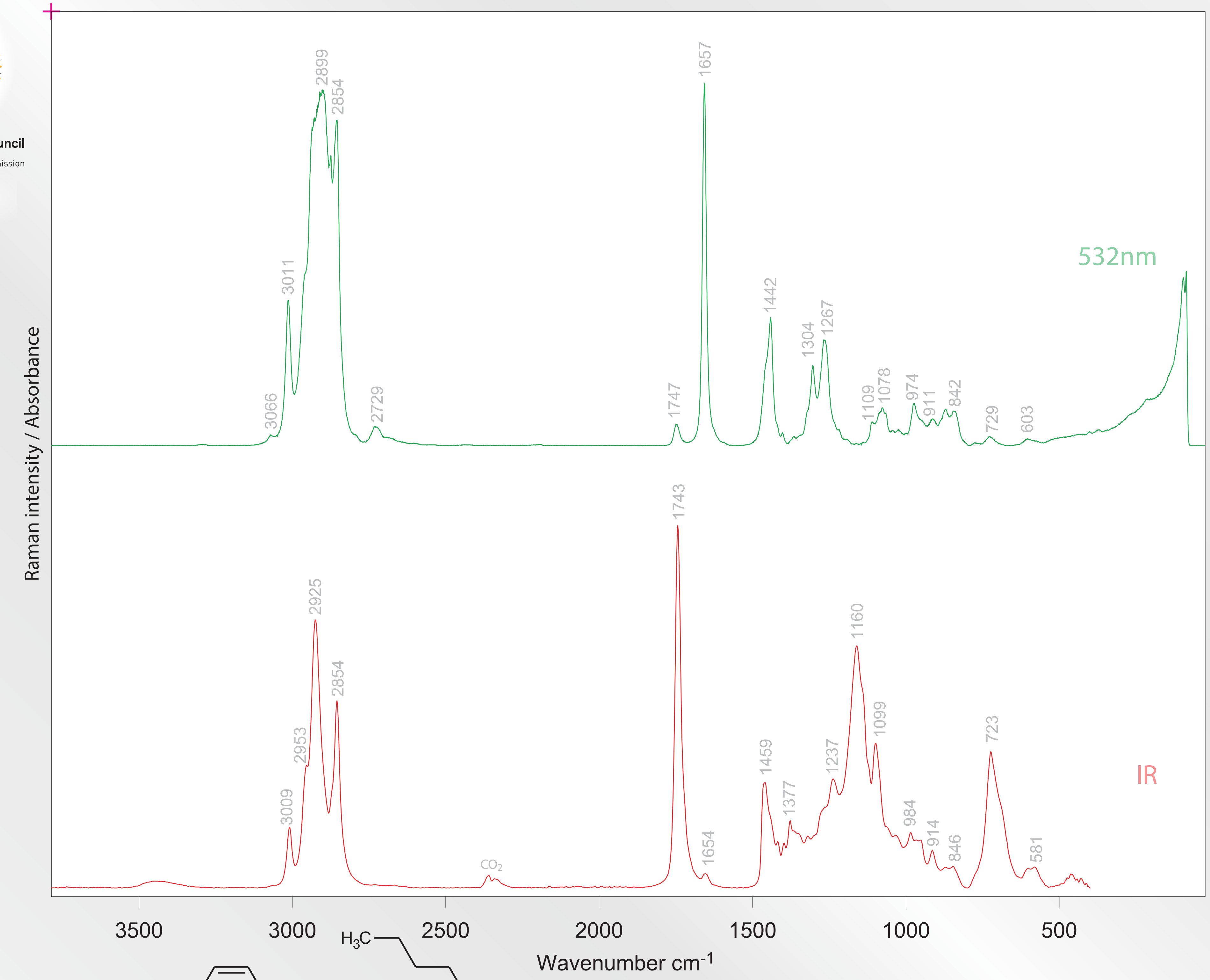

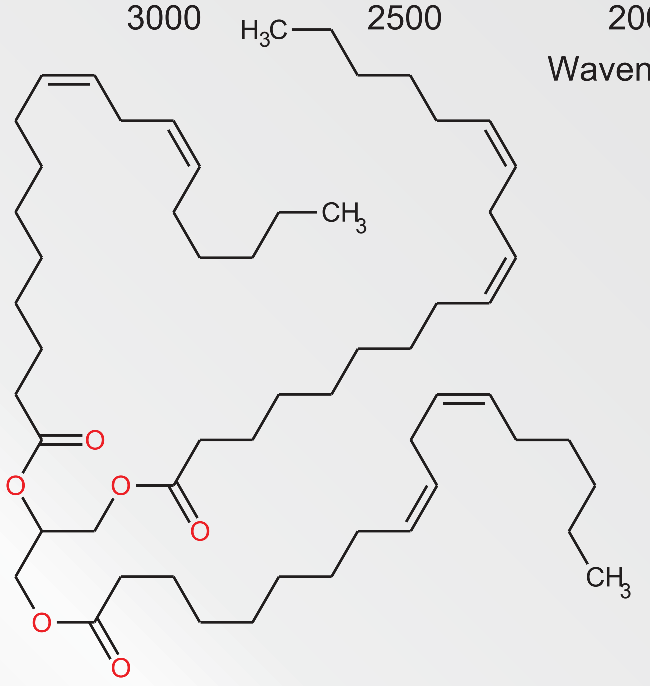

Sample:	T9517	Mode:	Vertex ATR
Producer:	Sigma	n_{Acc}:	32
Purity:	98%		
State:	liquid		
Temperature:	21°C		
measured in:	neat		

λ_{ex}:	532.001 nm
LPO:	33.3 mW
ν (Si):	520.67 cm^{-1}
t_{int}:	3.09756 s
Grating:	1800 g/mm
p_{Laser}:	0°
$p_{Spectrometer}$:	unpol
Objective:	20x air NA 0.4

IR	532
3443	
3057	3070
3009	3014
2953	***2957***
2925	***2935***
	2925
	2910
	2899
	2874
2854	2854
	2732
1743	1747
1654	***1656***
1459	1455
	1441
1418	1421
1397	1403
1377	
1366	1364
1350	
1320	1324
	1303
1275	
1261	1267
1237	
1160	1218
1143	
1122	
	1109
1099	
	1076
1061	1062
	1045
1034	
	1025
	1001
984	
966	973
952	
914	914
871	870
846	846
723	727
689	
602	605
581	
473	
463	
442	
429	
412	
	211
	96
	86

ν...stretch, δ...in-plane bend, γ...out-of-plane bend, τ...torsion, γ_r...rocking, γ_w...wagging, γ_t...twisting Φ...ring (Varsanyi), d...degenerate s...symmetric/scissoring, as... a(nti-)symmetric, ip...in-phase, op...out-of-phase

Peter Bock, 26.03.2018
