# Supplementary material for: Antifungal stilbene impregnation: transport and distribution on the micron-level
Source: Tree Physiol. 2018 Jul 10;38(10):1526–37. doi: 10.1093/treephys/tpy073 (PMC6198867; doi:10.1093/treephys/tpy073)
Supplement: Supplementary Data [file tpy073_si_felhofer_pine.docx]

***Tree Physiology* Supporting Information**

**Article title:**

Antifungal stilbene impregnation: transport and distribution on the micron-level

**Authors:**

Martin Felhofer^1^, Batirtze Prats-Mateu^1^, Peter Bock^1^, *Notburga Gierlinger^1^

**The following Supporting Information is available for this article:**

**Fig. S1 |** Sources of stilbenes from different plants.

**Fig. S2 |** Schematic illustration of the experimental procedure for the impregnation part.

**Fig. S3 |** Reference spectra related to Figure 4.

**Fig. S4 |** Impregnated spruce sapwood related to Figure 6.

**Table S1 |** Data overview of the pine sample tree

**Reference spectra:**

**Assignment Table S1 |** Pinosylvin (PS).

**Assignment Table S2 |** Pinosylvin monomethyl ether (PSMME).

**Assignment Table S3 |** Pinosylvin dimethyl ether (PSDME).

**Assignment Table S4 |** Resveratrol

**Assignment Table S5 |** Linoleic acid

**Assignment Table S6 |** Oleic acid

**Assignment Table S7 |** Abietic acid

**Assignment Table S8 |** Glycerol trilineolate


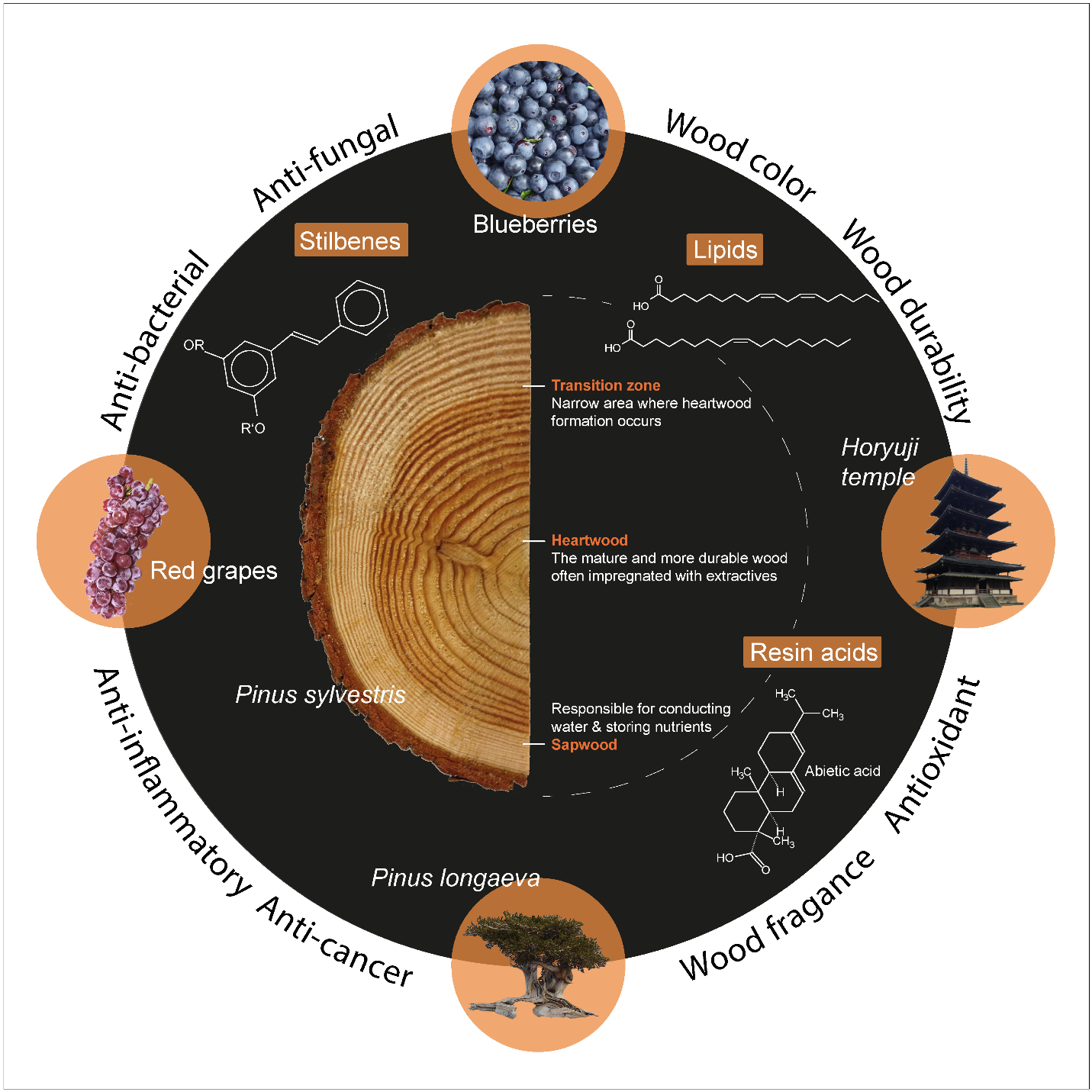


**Supporting Fig. S1 | Sources of stilbenes from different plants:**

Stilbenes are naturally produced and distributed in diverse plant families, including grape (Vitaceae), pine (Pinaceae), peanut (Fabaceae) and sorghum (Poaceae) ([Dubrovina and Kiselev, 2017](#_ENREF_3)). Development and transportation knowledge, but also the manipulation of heartwood extractives has the potential to improve wood properties and our basic understanding of heartwood formation. The xylem of a pine tree has three major parts: Sapwood, Heartwood and the Transition zone between. Photo credits: Blueberries photograph by Rosa-Maria Rinkl under a CC-BY-SA-4.0 license; Horyuji Temple photograph by [そらみみ](https://commons.wikimedia.org/wiki/User:%E3%81%9D%E3%82%89%E3%81%BF%E3%81%BF) under a CC-BY-SA-3.0; *Pinus Longaeva* photograph by Loren Reinhold under a PD-US-NPS license; Red grapes photograph by Bio06940 under a CC-BY-SA-3.0 license. Photo courtesy of Wikimedia Commons (<https://commons.wikimedia.org>).


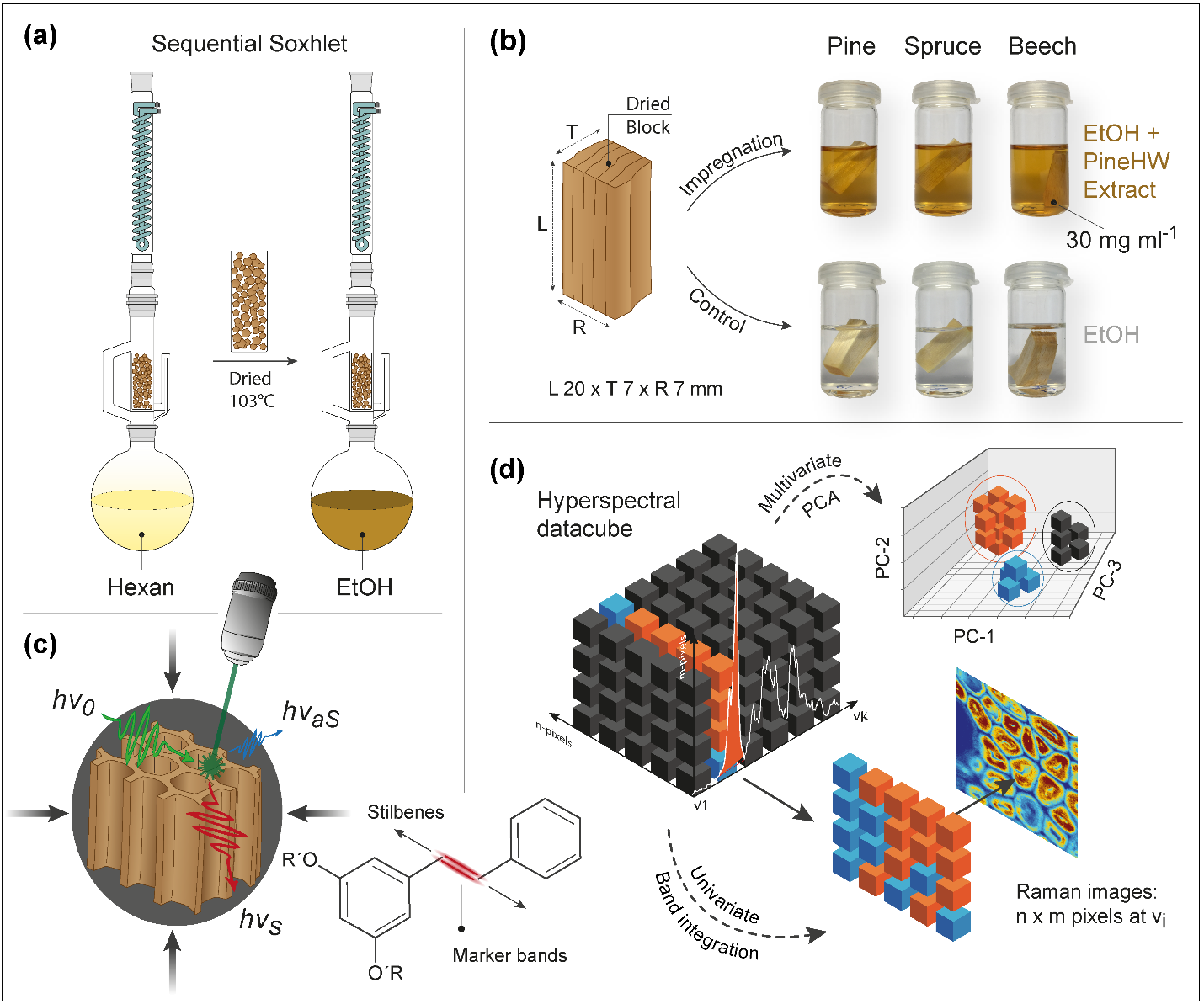


**Supporting Fig. S2 | Schematic illustration of the experimental procedure for the impregnation part:** **(a)** Sequential Soxhlet aperture with the cooler on top, first the hexane extract step to gain the lipophilic part and secondly the EtOH extracting step to ex-crude the hydrophilic part. **(b)** Impregnation of the kiln-dried wood blocks with an approximate size of longitudinal [L] 20 mm, tangential [T] 7 mm and radial [R] 7 mm. **(c)** Raman measurement of the impregnated wood with stilbenes represented by marker bands. **(d)** Gained images from the Raman hyperspectral data-cube by the univariate methods (band integration) as well as the multivariate method sketch of the PCA.


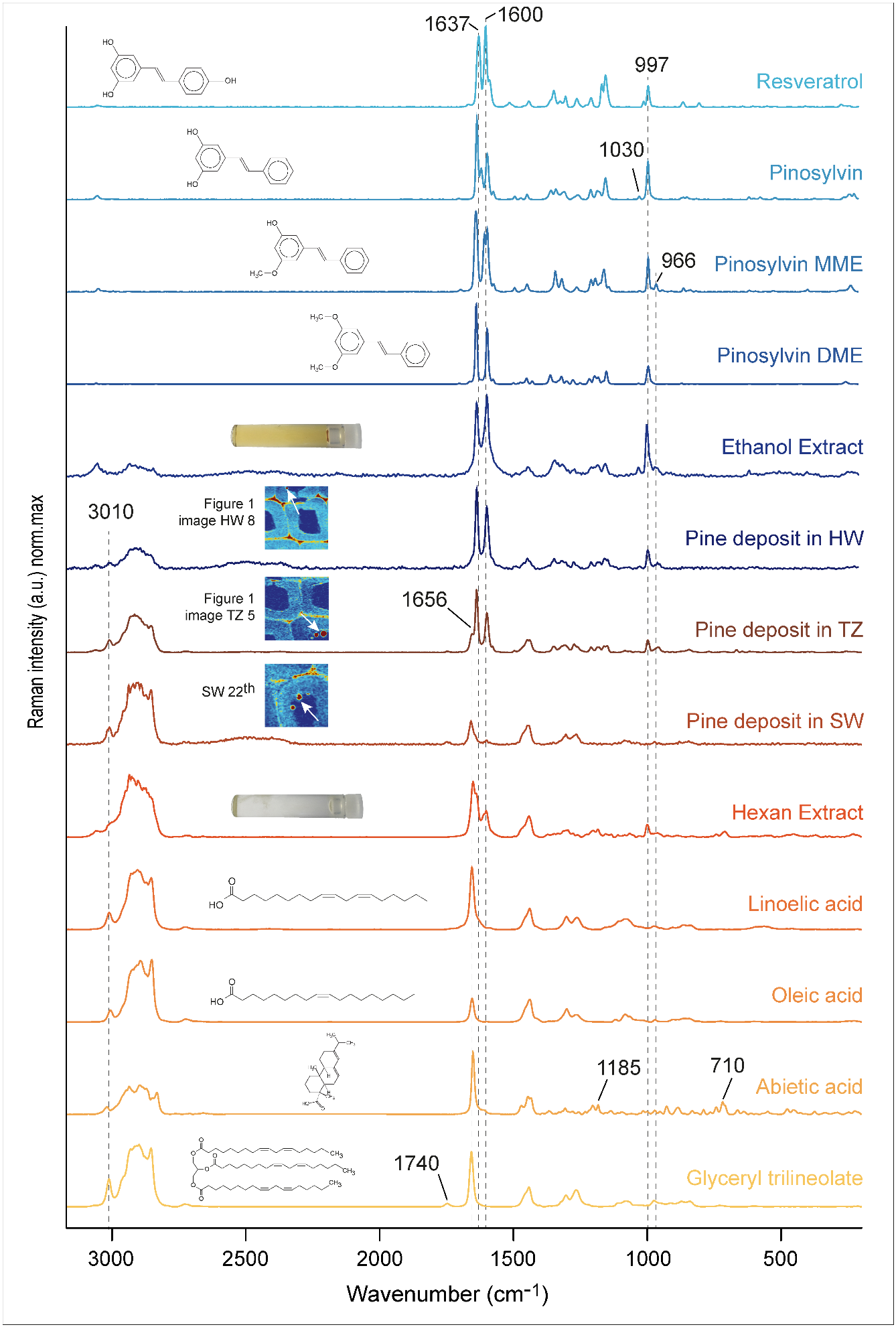


**Supporting Fig. S3 | Reference spectra related to Figure 4:**

The spectra are analyzed with OPUS, baseline corrected using concave rubberband (polynomial 6) and min. –max normalized in a frequency range from 200 to 3170 cm^-1^. Experimental set up: λ 532 nm, Laserpower 30 mW, Grating 600 g/mm. The Raman images show the deposits in the native pine wood (related to figure 2), marked with white arrows.


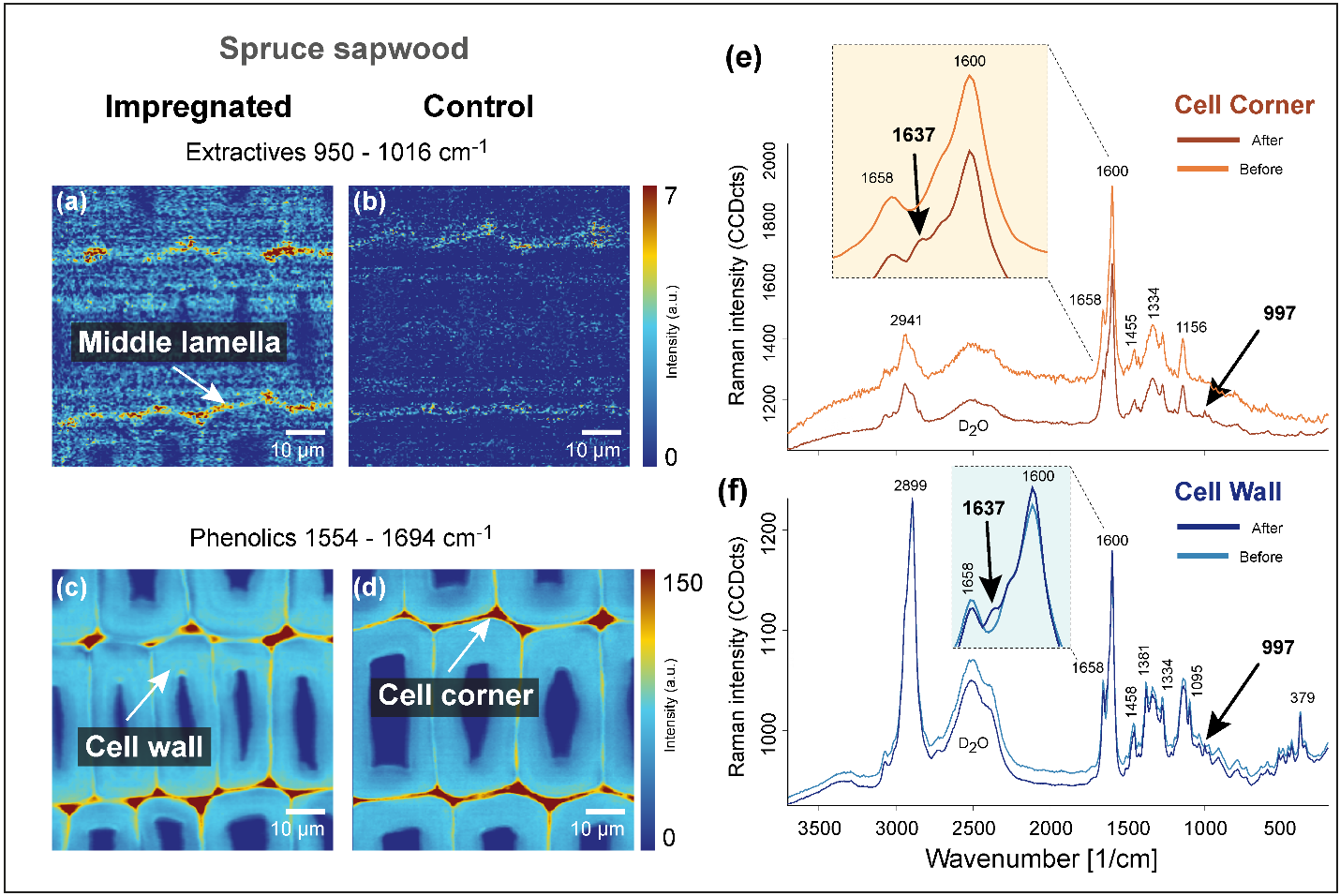


**Supporting Fig. S4 | Impregnated spruce sapwood related to Figure 6:**

**(a-d)** Intensity maps of the phenolics and extractives distribution for native spruce wood and after impregnation. **(a-b)** Raman integration maps for extractives from 950 to 1016 cm^-1^ reveal the higher impregnation of the cell corners and middle lamella with stilbene rich EtOH extract. **(c-d)** Lignin and extractives distribution within cell corner, cell wall and middle lamella by integrating from 1554-1692 cm^-1^. Experimental set up: Image size 70x70 µm^2^, λ 532 nm, Laserpower 30 mW, Integration time 0.08 s, Grating 600 g/mm. (e-f) Comparison of the Raman spectra of spruce cell corner and cell wall before and after impregnation. The two distinct Raman peaks (1637 and 997 cm^-1^) used to identify the impregnation is present in cell corners **(e)** and cell wall **(f)** as marked in the spectra. The spectra are analyzed with OPUS and cut from 200-3700 cm^-1^.

**Supporting Table S1 |** Data overview of the sampled Pinus sylvestris tree: Sampling height of the discs (Tree height), the age of the tree at a different height (Age), density of kiln dry samples (Density), moisture content determined with kiln dry method for heartwood- and sapwood (mc).

| Tree height (m) | Age (years) | Density _(hw)_ | Density _(sw)_ | m_c_ (hw.) | m_c_ (sw.) |
| --- | --- | --- | --- | --- | --- |
| 1.3 m | 62 | 519  kg m^-3^ | 609 kg m^-3^ | 30 % | 102 % |
| 6 m | 47 | 497  kg m^-3^ | 550  kg m^-3^ | 31.4  % | 119.2  % |
| 12 m | 29 | 425  kg m^-3^ | 481  kg m^-3^ | 30.8  % | 137.5  % |

Heartwood (hw), sapwood (sw), moister content fresh (m_c_), Density dried 103°C

**References**

AGARWAL, U. P., MCSWEENY, J. D. & RALPH, S. A. 2011. FT–Raman Investigation of Milled-Wood Lignins: Softwood, Hardwood, and Chemically Modified Black Spruce Lignins. *Journal of Wood Chemistry and Technology,* 31**,** 324-344.

BILLES, F., MOHAMMED-ZIEGLER, I., MIKOSCH, H. & HOLMGREN, A. 2002. Vibrational spectroscopic and conformational analysis of pinosylvin. *Journal of Physical Chemistry A,* 106**,** 6232-6241.

DUBROVINA, A. S. & KISELEV, K. V. 2017. Regulation of stilbene biosynthesis in plants. *Planta*.
